# Effects of *Sparganii Rhizoma* on Osteoclast Formation and Osteoblast Differentiation and on an OVX-Induced Bone Loss Model

**DOI:** 10.3389/fphar.2021.797892

**Published:** 2022-01-04

**Authors:** Sungyub Lee, Minsun Kim, Sooyeon Hong, Eom Ji Kim, Jae-Hyun Kim, Youngjoo Sohn, Hyuk-Sang Jung

**Affiliations:** Department of Anatomy, College of Korean Medicine, Kyung Hee University, Seoul, South Korea

**Keywords:** sparganii rhizoma, bone remodeling, osteoclast, osteoblast, ovariectomized

## Abstract

Postmenopausal osteoporosis is caused by an imbalance between osteoclasts and osteoblasts and causes severe bone loss. Osteoporotic medicines are classified into bone resorption inhibitors and bone formation promoters according to the mechanism of action. Long-term use of bisphosphonate and selective estrogen receptor modulators (SERMs) can cause severe side effects in postmenopausal osteoporosis patients. Therefore, it is important to find alternative natural products that reduce osteoclast activity and increase osteoblast formation. *Sparganii Rhizoma* (SR) is the dried tuberous rhizome of *Sparganium stoloniferum* Buchanan-Hamilton and is called “samreung” in Korea. However, to date, the effect of SR on osteoclast differentiation and the ovariectomized (OVX)-induced bone loss model has not been reported. *In vitro*, tartrate-resistant acid phosphatase (TRAP) staining, western blots, RT-PCR and other methods were used to examine the effect of SR on osteoclast differentiation and osteoblasts. *In vivo*, we confirmed the effect of SR in a model of OVX-induced postmenopausal osteoporosis. SR inhibited osteoclast differentiation and decreased the expression of TNF receptor-associated factor 6 (TRAF6), nuclear factor of activated T cells 1 (NFATc1) and c-Fos pathway. In addition, SR stimulates osteoblast differentiation and increased protein expression of the bone morphogenetic protein 2 (BMP-2)/SMAD signaling pathway. Moreover, SR protected against bone loss in OVX-induced rats. Our results appear to advance our knowledge of SR and successfully demonstrate its potential role as a osteoclastogenesis-inhibiting and osteogenesis-promoting herbal medicine for the treatment of postmenopausal osteoporosis.

## Introduction

Bone remodeling is maintained by a balance between bone resorption by osteoclasts and bone formation by osteoblasts (Matsuo and Irie, 2008). However, bone metabolic diseases such as osteoporosis and Paget’s disease occur when bone formation is reduced due to a decrease in the activity of osteoblasts. Additionally, excessive activity of osteoclasts is increased or when the two factors act at the same time (Feng and McDonald, 2011). Osteoporosis is known to be associated with several factors (menopause and aging), and it is characterized by excessive bone loss, weakening of bone microstructure and increased risk of fracture (Sozen et al., 2017). Osteoporotic medicines are classified into bone resorption inhibitors and bone formation promoters according to the mechanism of action. For the treatment of osteoporosis, bone resorption inhibitors such as bisphosphonate and selective estrogen receptor modulators (SERMs) are mainly used (Kennel and Drake, 2009; Gu et al., 2016). However, bone resorption inhibitors cannot treat osteoporosis that has already progressed. In addition, long-term use of bisphosphonate and SERMs can cause severe side effects in postmenopausal osteoporosis patients, such as osteonecrosis of the jaw, atrial fibrillation, abnormal vaginal bleeding, hot flashes and atrophic vaginitis (Bamias et al., 2005; Gu et al., 2016). Thus, the discovery of alternative natural products that decrease osteoclast activity and increase osteoblast formation with fewer side effects than the current medications is crucial.

Osteoclasts are multinucleated giant cells derived from mononuclear and macrophage precursor cells (Collin-Osdoby and Osdoby, 2012). Receptor activator of nuclear factor-κB ligand (RANKL) plays a critical role in the differentiation of osteoclast precursor cells into mature osteoclasts (Suda et al., 1999). On the surface of osteoclasts, the binding receptor activator of nuclear factor-κB (RANK) to RANKL activates TNF receptor-associated factor 6 (TRAF6) (Clohisy et al., 2003). Then, activation of TRAF6 induces nuclear factor-κB (NF-κB) and mitogen-activated protein kinase (MAPK). These signaling pathways activate transcription factors such as nuclear factor of activated T cells 1 (NFATc1) and c-Fos (Galibert et al., 1998; Boyle et al., 2003). NFATc1 is essential for osteoclast differentiation and regulates the expression of osteoclast-related genes (Takayanagi et al., 2002; Kim and Kim, 2014). Osteoblasts are derived from mesenchymal stem cells and large cells responsible for bone synthesis and mineralization. Bone morphogenetic protein 2 (BMP-2) and runt-related transcription factor 2 (RUNX-2) are important transcription factors for osteoblast differentiation and bone formation (Liu et al., 2007). These transcription factors upregulate the expression of osteogenic markers such as alkaline phosphatase (ALP), bone sialoprotein (BSP) and collagen type 1 (COL1) (Liu et al., 2007).

Korean medicine has recently been spotlighted as an alternative medicine for various diseases due to its few side effects and high therapeutic effects (Yuan et al., 2016). *Sparganii Rhizoma* (SR) is the dried tuberous rhizome of *Sparganium stoloniferum* Buchanan-Hamilton and is called “samreung” in Korea (Jia et al., 2021). SR has been used as a traditional Korean medicine to treat patients with gynecological diseases such as uterine fibroids, blood stasis and dysmenorrhea (Zhao et al., 2018). The chemical components of SR have been identified as phenylpropanoids (such as ferulic acid, *p*-coumaric acid, caffeic acid), flavonoids (such as kaempherol, rutin, formononetin), coumarins (such as sparstolonin B), volatile oils (β-pinene, eucalyptol, myrtenol) and others (Jia et al., 2021). Among them, kaempferol, rutin, formononetin, ferulic acid, p-coumaric acid and caffeic acid were previously found to inhibit osteoclast differentiation (Kyung et al., 2008; Sagar et al., 2016; Doss et al., 2018; Kim et al., 2018; Liu et al., 2020; Ekeuku et al., 2021). Inflammation has been associated with metabolic bone diseases such as postmenopausal osteoporosis and rheumatoid arthritis. Previous studies have shown that estrogen deficiency promotes bone loss by inducing an increase in inflammatory cytokines such as IL-6 and TNF-α. The pro-inflammatory cytokines TNF-α and IL-6 are potent inducers of bone resorption. Previous studies have confirmed that TNF-α can promote RANKL-induced osteoclast formation *in vitro*. IL-1 promotes multinucleation of osteoclast precursors and enhances the ability of mature multinucleated cells to resorb bone. Therefore, it is suggested that increased expression of inflammatory cytokines is associated with osteoporosis (Ginaldi et al., 2005). Among the components of *p*-coumaric acid, caffeic acid, kaempferol, rutin, formononetin, and sparstolonin B have been proven to have anti-inflammatory effects through previous studies (Guardia et al., 2001; Pragasam et al., 2013; Kadioglu et al., 2015; Luo et al., 2019; Yepuri et al., 2019; Zielinska et al., 2021). Therefore, it was expected that SR would be effective for postmenopausal osteoporosis because of the pharmacological effects of SR, which have been traditionally used for female diseases, and the inhibition of osteoclast differentiation and anti-inflammatory effects of SR components. However, to date, the effect of SR on osteoclast differentiation and an ovariectomized (OVX)-induced bone loss model has not been reported. Therefore, this study was performed to explore the effects of SR on osteoclast differentiation and osteoblasts as well as the underlying mechanism and OVX-induced bone loss models.

## Materials and Methods

### Reagents

Dulbecco’s modified Eagle’s medium (DMEM) was obtained from Welgene (Daejeon, Korea). Minimum essential medium Eagle alpha-modification (α-MEM), penicillin/streptomycin (P/S), Dulbecco’s phosphate buffered saline (DPBS) and normal serum for immunohistochemistry were purchased from Gibco (Gaithersburg, MD, United States). Fetal bovine serum (FBS) was supplied by Atlas Biologicals (Fort Collins, CO, United States). RANKL was purchased from Peprotech (London, United Kingdom). Cell Counting Kit-8 (CCK-8; WST-8; 2-(2-methoxy-4-nitrophenyl)-3-(4-nitrophenyl)-5-(2,4-disulfophenyl)-2H-tetrazolium, monosodium salt) was purchased from Dojindo Molecular Technologies, Inc. (Japan, Kumamoto). Osteo assay stripwell plates were purchased from Corning, Inc. (New York, NY, United States). Tartrate-resistant acid phosphatase (TRAP) kits, 4′,6-diamidino-2-phenylindol (DAPI), β-glycerophosphate and ascorbic acid, 17β-estradiol (E_2_) and alendronate (ALN) were obtained from Sigma-Aldrich; Merck KGaA (Darmstadt, Germany). Acti-stain™ 488 Fluorescent Phalloidin was purchased from Cytoskeleton, Inc. (Denver, CO, United States). Primary antibodies against anti-TRAF6 (TRAF6; cat no: sc- 8409), anti-Lamin B (Lamin B; cat no: sc- 374015), anti-β-actin (Actin; cat no: 8432) and anti-c-Fos (c-Fos; cat no: sc-447) were purchased from Santa Cruz Biotechnology, Inc. (Santa Cruz, CA, United States). Anti-NFATc1 (NFATc1; cat no: 556602) was purchased from BD Pharmingen, Inc. (San Diego, CA, United States). Anti-RUNX-2 (RUNX-2; cat no: ab), anti-Osterix (Osterix; cat no: ab209484) and anti-SMAD 1/5/9 (SMAD1/5/9; cat no: ab80255) were purchased from Abcam (Cambridge, MA, United States). Anti-phosphorylated (p)-extracellular signal-regulated kinase (p-ERK; cat no: 4370S), anti-ERK (ERK; cat no: 4695S), anti-p-c-Jun N-terminal kinase (p-JNK; cat no: 4668S), anti-JNK (JNK; cat no: 9258S), anti-p-p38 (p-p38; cat no: 4511S), anti-p38 (p38; cat no: 9212L), anti-p-NF-κB (p-NF-κB; cat no: 3032S), anti-NF-κB (NF-κB; cat no: 8242S), anti-p-IκB (p-IκB; cat no: 2859S), anti-IκB (IκB; cat no: 4814S) and anti-phospho-SMAD1/5 (p-SMAD1/5; cat no: 9516S) was purchased from Cell Signaling Technology, Inc. (Danvers, MA, United States), and secondary antibodies were purchased from Jackson ImmunoResearch Laboratories, Inc. (West Grove, PA, United States). Masson-goldner trichrome kit was purchased from BioGnost (cat no: MGT-100T, Zagreb, Croatia, EU). Proteinase K was supplied by Thermo Fisher Scientific (Waltham, MA, United States). Biotinylated secondary antibody, ABC HRP Kit (Peroxidase, Standard) and 3,3′-diaminobenzidine (DAB) solution were purchased from Vector Labs (Burlingame, CA, United States).

### Preparation of *Sparganii Rhizoma* Ethanol Extract

SR was purchased from Omniherb Co. (Yeoungcheon, Korea). A total of 150 g of the dried herbs was extracted in 80% ethanol for 2 weeks. The extracts were filtered using filter paper (no. 3; Whatman PLC; GE Healthcare). Then, the ethanol extract was evaporated using a vacuum concentrator. Then, the extract was filtered and evaporated using a vacuum to yield the powder (dried powder: 5.5 g), and the SR extract yield was 3.7%.

### Cell Culture and Cell Cytotoxicity Assay

The RAW 264.7 macrophage line was obtained from the Korean Cell Line Bank (KCLB No. 40071; Seoul, Korea). RAW 264.7 cells were cultured in DMEM containing 10% FBS and 1% P/S in a 5% CO_2_ incubator (Thermo Fisher; Waltham, MA) at 37°C. For determination of the effect of SR on RAW 264.7 cell cytotoxicity, cell viability was measured using the Cell Counting Kit-8 (CCK-8) assay. For the RAW 264.7 cell cytotoxicity assay, RAW 264.7 cells were seeded into 96-well plates at a density of 5 × 10^3^ cells/well at 37°C for 24 h. Then, the cells were treated with SR (125, 250, 500, 1,000 μg/ml) at 37°C for 1 day. For the osteoclast cytotoxicity assay, RAW 264.7 cells were seeded in 96-well plates at a density of 5 × 10^3^ cells/well at 37°C for 24 h. Then, the cells were treated with SR (125, 250, 500, 1,000 μg/ml) and kaempferol (209.5 ng/ml and 419 ng/ml) at 37°C for 5 days. The cytotoxicity of RAW 264.7 cells and osteoclasts were measured using the Cell Counting Kit-8 (CCK-8) assay. Thereafter, we added 10 μL of CCK-8 solution to each well, and the cells were incubated at 37°C for 2 h. At the end of the experiment, the optical densities (ODs) at 405 nm were detected using an enzyme-linked immunosorbent assay (ELISA) reader (VersaMax microplate reader; Molecular Devices, LLC).

MC3T3-E1 subclone 4 cells were obtained from the American Type Culture Collection (No. ATCC-CRL-2593, Manassas, VA). MC3T3-E1 cells were cultured in α-MEM (without ascorbic acid) containing 10% FBS and 1% P/S in a 5% CO_2_ incubator at 37°C. For determination of the effect of SR on MC3T3-E1 cell cytotoxicity assay, MC3T3-E1 cells were seeded in a 96-well plate at a density of 1 × 10^4^ cells/well overnight in a 5% CO_2_ incubator at 37°C for 24 h, and then, the cells were treated with SR at various concentrations (125, 250, 500, 1,000 μg/ml) for 3, 7 and 14 days. For determination of the effect of kaempferol on MC3T3-E1 cell cytotoxicity assay, MC3T3-E1 cells were seeded in a 96-well plate at a density of 1 × 10^4^ cells/well overnight in a 5% CO_2_ incubator at 37°C for 24 h, and then, the cells were treated with kaempferol at various concentrations (41.9 ng/ml and 83.8 ng/ml) for 3 and 7 days. Thereafter, we added 10 μL of CCK-8 solution to each well, and the cells were incubated at 37°C for 2 h. Then, cell cytotoxicity was measured using enzyme-linked immunosorbent assays (ELISA; VersaMax, Molecular Devices, LLC, Sunnyvale, CA). Then, cell cytotoxicity was measured using the CCK-8 assay, and absorbance was detected with a microplate reader at 405 nm. Cytotoxicity is expressed as a percentage compared to that of the normal group, and values less than 90% were considered to indicate cytotoxicity.

### TRAP Staining and TRAP Activity

To determine the effect of SR on osteoclast differentiation, we seeded RAW 264.7 cells in 24-well plates at a density of 1.5 × 10^4^ cells/well and cultured them at 37°C for 24 h. Then, RAW 264.7 cells were replaced with α-MEM containing RANKL (100 ng/ml) and SR (125, 250, 500, 1,000 μg/ml) at 37°C for 5 days. The differentiation medium was replaced every 2 days. In order to compare the inhibitory effect of SR and kaempferol on osteoclast differentiation, RAW 264.7 cells were seeded in 24-well plates at a density of 1.5 × 10^4^ cells/well and cultured them at 37°C for 24 h. Then, the medium was replaced with RANKL (100 ng/ml) and SR (500 and 1,000 μg/ml) or kaempferol (209.5 ng/ml and 419 ng/ml) at 37°C for 5 days. After the end of osteoclast differentiation, the cells were fixed with 10% formaldehyde at room temperature for 10 min. Then, tartrate-resistant acid phosphatase (TRAP) staining was performed using a TRAP kit. The stained cells were imaged with an inverted microscope (Olympus, Tokyo, Japan). Cells with more than three nuclei or more nuclei were considered TRAP-positive osteoclasts.

To investigate the effect of SR on TRAP activity in differentiation medium, we transferred 50 μL of differentiation medium to a new 96-well plate. Thereafter, an equal volume of TRAP solution [4.93 mg p-nitrophenyl phosphate (PNPP) in 750 ml of 0.5 M acetate solution and 150 ml of tartrate acid solution] was added to each well and cultured at 37°C for 1 h. Thereafter, the reaction was stopped using 0.5 M NaOH, and the level of TRAP activity was measured using an ELISA reader at 405 nm.

### Pit Formation Assay and F-Actin Ring Staining

For analysis of the effect of SR on bone resorption, RAW 264.7 cells were seeded in osteoassay surface multiple well plates at a density of 5 × 10^3^ cells/well and cultured for 24 h. The cells were treated with replacement α-MEM containing RANKL (100 ng/ml) and SR (125, 250, 500, 1,000 μg/ml) for 5 days. The differentiation medium was replaced every 2 days. Afterward, the cells were washed and removed using deionized water with 4% sodium hypochlorite. The pit area was captured with an inverted microscope and measured using ImageJ software, and pit area measurements expressed the absorption area as a percentage of the total area.

For determination of the effects of SR on F-actin ring formation, RAW 264.7 cells were seeded in a 96-well plate at a density of 5 × 10^3^ cells/well and cultured for 24 h. RAW 264.7 cells were stimulated with RANKL (100 ng/ml) and SR (125, 250, 500, 1,000 μg/ml) at 37°C for 5 days. The cells were washed with phosphate-buffered saline (PBS) and fixed with 4% paraformaldehyde for 20 min. Then, the cells were permeabilized for 5 min with 0.1% Triton X-100. The cells were stained for Acti-stain™ 488 Fluorescent Phalloidin and DAPI for nuclei at room temperature in the dark for 30 min. Images were captured using fluorescence microscopy (Cellena, Logos Biosystems; magnification, ×200) and counted using ImageJ software version 1.46 (National Institutes of Health, Bethesda, MD).

### Western Blotting Analysis

To the examination of the effect of SR on the expression of TRAF6/MAPK/NF-kB signaling pathway, RAW 264.7 cells were seeded in a 60 π dish at a density of 2 × 10^6^ cells/well and cultured for 24 h. The cells were treated with α-MEM containing RANKL (100 ng/ml) and SR (125, 250, 500, 1,000 μg/ml) for 6 h. For determination of the effect of SR on the expression of NFATc1, c-Fos and MMP-9, RAW 264.7 cells were seeded in a 60 π dish at a density of 5 × 10^5^ cells/well and cultured for 24 h. The cells were treated with α-MEM containing RANKL (100 ng/ml) and SR (125, 250, 500, 1,000 μg/ml) or kaempferol (209.5 ng/ml and 419 ng/ml) for 1 day. For determination of the effect of SR on the expression of osteoblast differentiation transcription factors, MC3T3-E1 cells were seeded in a 60 π dish at a density of 5 × 10^5^ cells/well overnight in a 5% CO_2_ incubator at 37°C for 24 h, and then, the cells were treated with SR (25, 50, 100, 200 μg/ml) or kaempferol (41.9 ng/ml and 83.8 ng/ml) for 2 days. Total protein was extracted using RIPA buffer (composition: 50 mM Tris-Cl, 150 mM NaCl, 1% NP-40, 0.5% sodium deoxycholate and 0.1% SDS). Then, the extracts were centrifuged at 4°C for 20 min (2,000 rpm). Nuclear protein was extracted using a nuclear extraction kit (Thermo Fisher Scientific, Waltham, MA, United States).

The supernatants were collected, and protein (30 μg) was quantized by using a BCA protein assay kit. Thirty micrograms of protein were then separated using 10% SDS-PAGE and transferred onto a nitrocellulose membrane for 1 h. Subsequently, the membrane was blocked with 5% skim milk in TBST (containing 0.1% Tween 20) for 1 h at room temperature and incubated with primary antibodies at 4°C overnight. The primary antibodies used were as follows: TRAF6 (dilution; 1:1,000), p-NF-κB (dilution; 1:1,000), NF-κB (dilution; 1:1,000), p-IκB (dilution; 1:1,000), IκB (dilution; 1:1,000), Lamin B (dilution; 1:1,000), p-ERK (dilution; 1:1,000), ERK (dilution; 1:1,000), p-JNK (dilution; 1:1,000), JNK (dilution; 1:1,000), p-p38 (dilution; 1:1,000), p38 (dilution; 1:1,000), NFATc1 (dilution; 1:1,000), c-Fos (dilution; 1:200), β-actin (dilution; 1: 1,000), RUNX-2 (dilution; 1: 1,000), Osterix (dilution; 1: 1,000), SMAD1/5/9 (dilution; 1: 1,000) and p-SMAD1/5 (dilution; 1: 1,000). After 24 h, the membranes were washed three times with TBST and incubated with secondary antibodies at 37°C for 1 h. Then, the band was detected using electrochemiluminescence (ECL) solution. The protein levels were quantified by using ImageJ software version 1.46, and the resulting bands were normalized using a loading control.

### Real-Time Polymerase Chain Reaction (RT-PCR)

For determination of the effect of SR on osteoclast-related genes, RAW 264.7 cells were seeded in 6-well plates at a density of 2 × 10^3^ cells/well and cultured for 24 h. The cells were treated with α-MEM containing RANKL (100 ng/ml) and SR (125, 250, 500, 1,000 μg/ml) for 4 days. The differentiation medium was replaced every 2 days. Total RNA was extracted from TRIzol® reagent according to the manufacturer’s instructions (Invitrogen, Carlsbad, CA, United States). Total RNA (2 μg) was quantized using a NanoDrop 2000 instrument (Thermo Scientific, United Kingdom). cDNA reverse transcription was performed using a reverse transcription kit (Invitrogen; Thermo Fisher Scientific, Inc.). PCR was performed using a C1000 Touch™ Thermal Cycler (Bio-Rad Laboratories, Inc.). PCR conditions were as follows: 94°C for 1 min (denaturation), 55–58°C for 30 s (annealing) and 72°C for 1 min (extension). The primers used were as Table 1. PCR products were separated by 1.2% agarose gel. The band was quantified by using ImageJ software version 1.46, and the resulting products were normalized using β-actin.

**TABLE 1:** Primer sequences for RT–PCR analysis.

Genes	Primer sequence	Accession no.	Cycle	Temperature
*Nfatc1* (NFATc1)	F: TGC TCC TCC TCC TGC TGC TC	NM_198,429.2	32	58
	R: CGT CTT CCA CCT CCA CGT CG			
*Fos* (c-Fos)	F: ATG GGC TCT CCT GTC AAC AC	NM_010234.3	33	58
	R: GGC TGC CAA AAT AAA CTC CA			
*Mmp9* (MMP-9)	F: CGA CTT TTG TGG TCT TCC CC	NM_013599.4	30	58
	R: TGA AGG TTT GGA ATC GAC CC			
*Tnfrsf11a* (RNAK)	F: AAA CCT TGG ACC AAC TGC AC	NM_009399.3	32	53
	R: ACC ATC TTC TCC TCC CHA GT			
*Acp5* (TRAP)	F: ACT TCC CCA GCC CTT ACT ACC G	NM_007388.3	30	58
	R: TCA GCA CAT AGC CCA CAC CG			
*Ca2* (CA2)	F: CTC TCA GGA CAA TGC AGT GCT GA	NM_001357334.1	32	58
	R: ATC CAG GTC ACA CAT TCC AGC A			
*Oscar* (OSCAR)	F: CTG CTG GTA ACG GAT CAG CTC CCC AGA	NM_001290377.1	35	53
	R: CCA AGG AGC CAG AAC CTT CGA AAC T			
*Atp6v0d2* (ATP6v0d2)	F: ATG GGG CCT TGC AAA AGA AAT CTG	NM_175,406.3	30	58
	R: CGA CAG CGT CAA ACA AAG GCT TGT A			
*Dcstamp* (DC-STAMP)	F: TGG AAG TTC ACT TGA AAC TAC GTG	NM_001289506.1	40	63
	R: CTC​GGT​TTC​CCG​TCA​GCC​TCT​CTC			
*Actb* (β-actin)	F: TTC TAC AAT GAG CTG CGT GT	NM_008084.3	30	58
	R: CTC ATA GCT CTT CTC CAG GG			

NFATc1, nuclear factor of activated T cells 1; MMP-9, matrix metalloproteinase-9; RANK, receptor activator of nuclear factor-κB; TRAP, tartrate-resistant acid phosphatase; CA2, carbonic anhydrase II; OSCAR, osteoclast-associated receptor; ATP6v0d2, ATPase H + Transporting V0 Subunit D2; DC-STAMP, dendritic cell-specific transmembrane protein.

### Alizarin Red S Staining

For determination of the effect of SR on osteoblast differentiation, MC3T3-E1 cells were seeded in 24-well plates at a density of 1 × 10^4^ cells/well and cultured for 24 h. Cells were treated in osteogenic medium (α-MEM without ascorbic acid supplemented with 10 mM β-glycerophosphate and 25 μg/ml ascorbic acid) and SR (25, 50, 100, 200 μg/ml) for 14 days. The cells were washed three times with PBS and fixed with 80% Et-OH for 1 h at 4°C. For Alizarin Red S staining, the cells were rinsed with distilled water (D.W) and stained with Alizarin Red solution for 5 min at room temperature, and images were captured using a camera. For quantification of the Alizarin Red S staining, 10% (v/w) cetylpyridinium chloride in sodium phosphate was added to each well and extracted for 10 min. The extract was measured using an ELISA reader at 570 nm.

### Experimental Animals and Treatment

Eleven-week-old female Sprague Dawley (SD) rats were purchased from Nara Biotech (230–250 g; Seoul, Korea) and housed at 22 ± 2°C and a relative humidity of 53–55% under a 12 h light-dark cycle. Rats were placed in separate cages and provided *ad libitum* access to food and water. All animal experiments were approved by the Committee of the Kyung Hee University Laboratory Animal Center (approval number: KHSASP-21-185). For determination of the effect of SR on postmenopausal osteoporosis, OVX osteoporosis was induced. All animals were acclimatized for 1 week before surgery. For surgery, the animals were deeply anesthetized by 100% oxygen and 5% isoflurane. Both ovaries were removed in animals in the OVX group, and animals in the sham groups did not undergo removal of the ovaries and received the same stress. During the surgery, the isoflurane concentration was adjusted to 2–3%. After wound closure, the animals received intraperitoneal administration of gentamicin (4 mg/kg) for 3 postsurgical days.

Afterward, the animals were randomized into the following six groups (*n* = 8 each): sham group (sham operation; oral administration of deionized water (D.W)), OVX group (OVX-induced; oral administration of D. W), E_2_ group (OVX-induced; oral administration of 100 μg/kg E_2_), ALN group (OVX-induced; oral administration of 3 mg/kg ALN), SR-L group (OVX-induced; oral administration of 4.92 mg/kg SR) and SR-H group (OVX-induced; oral administration of 31.48 mg/kg SR). Doses for SR-L and SR-H were calculated based on the following theory: according to Korean medicine, a single dose of 8 g is recommended based on an average adult weight of 60 kg. Therefore, SR required 4.92 mg/kg. The animals in the SR-L group were treated with 4.92 mg/kg. Rats are known to have a metabolism 6.4 times faster than humans (Tschop et al., 2011; Nair and Jacob, 2016) Based on these results, the SR-H group was treated with a concentration 6.4 times higher than that of the SR-L group. Therefore, the SR-H group was treated with 31.48 mg/kg. E_2_, ALN and SR were dissolved in vehicle administered for 8 weeks, and animal weight was measured once a week. The humanitarian termination points were as follows: 1) weight loss of more than 20% compared to that of the other rats; 2) difficulty eating due to uncomfortable walking; 3) severe infection and bleeding at the surgical site; 4) vomiting and hemoptysis; no animals exhibited abnormal behavior during the experiment. After 8 weeks, the rats were anesthetized with 100% oxygen and 5% isoflurane. For all animal sacrifices, lethal cardiac puncture up to 10 ml was performed (Beeton et al., 2007). After confirming that the heart had completely stopped, we performed cervical dislocation. Then, uterine samples were collected. We fixed the femur samples in 10% neutral buffered formalin (NBF). Blood samples were collected from the SD rats after sacrifice and centrifuged at 29,739 xg for 10 min.

### Serum Levels of TRAP, ALP, AST and ALT

The extracted blood was stored at room temperature for 15 min and then centrifuged at 29,739 xg for 10 min to separate the serum. The blood sample was stored at −4°C until the experiment. Serum TRAP activity was assessed in the same manner as described in a previous experimental method, and the serum expression of alkaline phosphatase (ALP), aspartate aminotransferase (AST) and alanine transferase (ALT) were performed by DKkorea (Seoul, Korea).

### Micro-CT Analysis

The femurs were analyzed with a microcomputed tomography (micro-CT, SkyScan1176, Skyscan, Kontich, Belgium) system. The scanning parameters were set as follows: a 50 kV/200 μA, 8.9 μm pixels, an aluminum (Al) filter of 0.5 mm, and a 180° rotation angle with rotation steps of 0.4°. Bone microstructure, such as bone mineral density (BMD), trabecular bone volume (BV/TV), trabecular thickness (Tb.Th), trabecular number (Tb.N) and trabecular separation (Tb.sp), was analyzed with NRecon software (SkyScan version 1.6.10.1; Bruker Corporation, Billerica, MA).

### Hematoxylin and Eosin (H&E) Staining, TRAP Staining and Masson-Goldner’s Trichrome Staining

The femurs were decalcified with 10% ethylenediaminetetraacetic acid (EDTA) for 3 weeks, embedded in paraffin, and then sectioned using a microtome (5 µm-thick; Carl Zeiss AG). Tissue sections were mounted on a slide dryer for 1 day. H&E staining was performed to measure the trabecular area. The trabecular areas were captured in H&E-stained sections under a light microscope (Olympus Corporation, magnification, ×100) and its was measured with ImageJ version 1.46. To confirm the bone resorption and bone formation parameters in femoral tissues, we stained them using the TRAP and masson-goldner’s trichrome staining kit according to the manufacturer’s protocol. The stained tissues were captured under a light microscope (Olympus Corporation, magnification, ×100 and ×200). Bone resorption parameters such as osteoclast surface per bone surface (Oc.S/BS) and osteoclast number per bone surface (Oc.N/BS) and bone formation parameters such as Osteoblast surface per bone surface (Ob.S/BS), the osteoblast number per bone surface (Ob.N/BS) were measured with ImageJ version 1.46.

### Immunohistochemical (IHC) Staining

For analysis of IHC staining, the tissues were deparaffinized by xylene. In addition, 0.3% hydrogen peroxide was used for 15 min to block endogenous peroxidase. Proteinase K (0.4 mg/ml) was used for antigen retrieval at 37°C for 30 min. After washes in PBS, section tissues were incubated with normal serum for 1 h and incubated with primary antibodies against CTK (1:100), NFATc1 (1:100) and BMP-2 (1:100) at 4°C overnight. Then, the tissues were washed with PBS and incubated with secondary antibodies for 1 h. The tissues were incubated with an ABC kit for 30 min, and the reaction was visualized with DAB solution. Counterstaining was performed with hematoxylin. The positive area was captured in the IHC staining sections under a light microscope (Olympus Corporation, magnification, ×100 and ×200).

### Liquid Chromatography–Mass Spectrometry (LC-MS) Analysis

Kaempferol (HPLC purity>97%; cat. no. 60010; Sigma-Aldrich; Merck KGaA) is a well-known flavonoid compound in SR (Wang et al., 2012; Wang et al., 2013). A Waters e2695 system (Milford, MA, United States) equipped with an ACQUITY QDa Detector was used to analyze the sample and kaempferol. Separation was performed on an Xbridge-C18 with a C18 guard column (250 × 4.6 mm; 5 μm; Waters Corporation). The mobile phase consisted of acetonitrile (A) and H_2_O with 1% acetic acid (B) and was run at 25°C for 40 min at a flow rate of 0.4 ml/min. The system was run with a gradient program: 0–40 min, 5–80% B. The sample injection volume was 10 μL. The mass spectrometer was fitted to an atmospheric pressure electrospray ionization (ESI) source operated in positive ion mode. The electrospray capillary voltage was set to 0.8 kV, and the cone voltage was 15 V. MS data were acquired in scan mode (mass range m/z 150–650).

### Statistical Analyses

All quantitative data are presented as the mean ± SD values of three experiments. Differences between the control and treatment groups were analyzed using one-way ANOVA after Dunnett’s post hoc tests. Statistical significance was considered at *p* < 0.05. Statistical analysis was performed using GraphPad PRISM (version 5.01, GraphPad Software, Inc., San Diego, United States).

## Results

### *Sparganii Rhizoma* Decreased the Number of TRAP-Positive Cells and TRAP Activity

**FIGURE 1:**
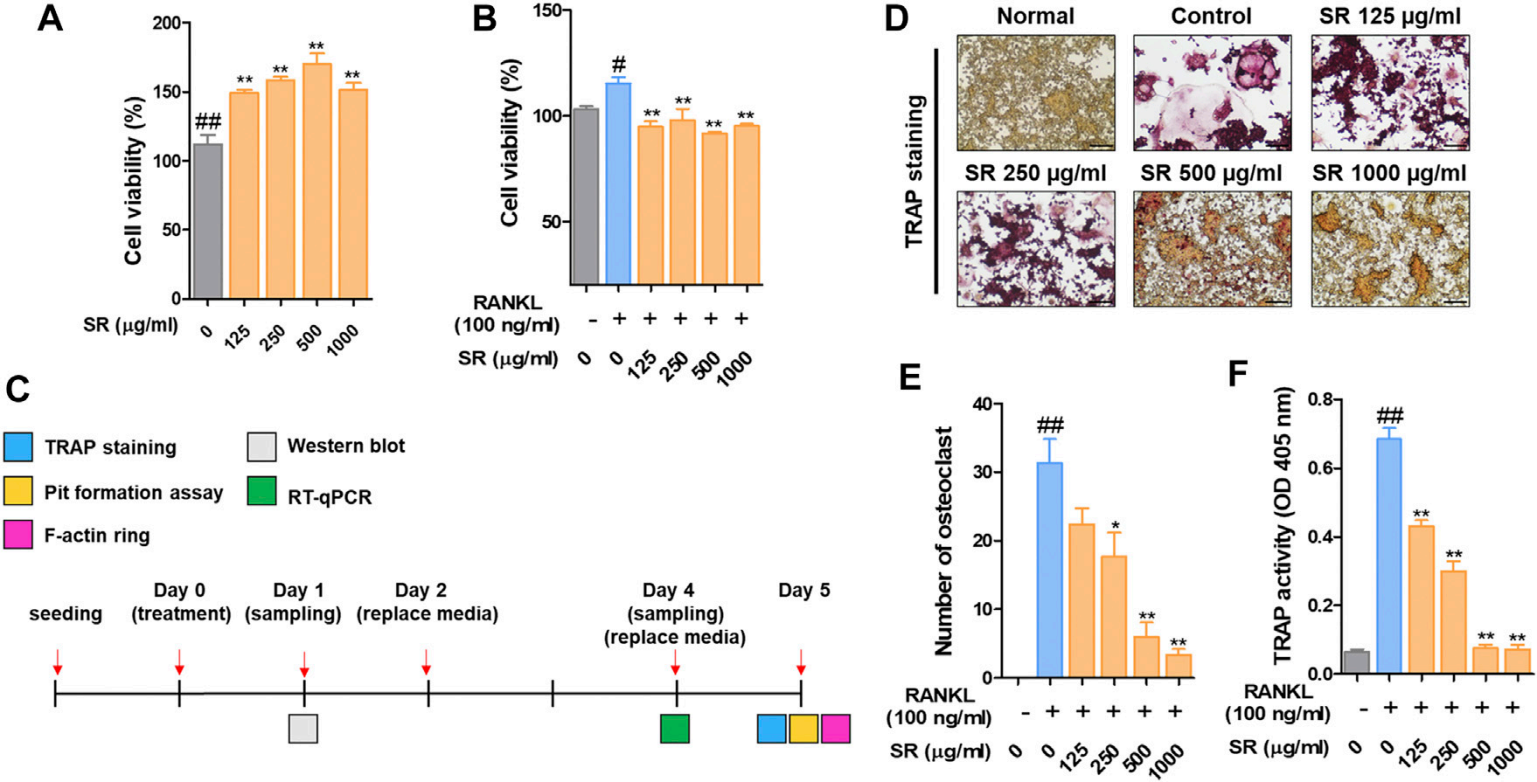
The effects of SR on osteoclast differentiation and bone resorption in RANKL-induced RAW 264.7 cells. **(A)** RAW 264.7 cell viability was measured using a CCK-8 assay kit. **(B)** The effects of SR (0, 125, 250, 500, 1,000 μg/ml) on the viability of osteoclasts induced by RANKL using a CCK-8 assay kit. **(C)** *In vitro* experimental design to investigate the effect of SR on osteoclast differentiation. **(D)** TRAP-positive cells were stained using a TRAP kit. **(E)** A number of TRAP-positive cells with >3 nuclei were counted using an inverted microscope (magnification, ×100). **(F)** TRAP activity was measured using an ELISA reader. Data are presented as the mean ± SD of three independent experiments. Statistical analysis was performed using one-way ANOVA followed by Dunnett’s post hoc test. ^#^ *p* < 0.05, ^##^ *p* < 0.01 vs. the normal group (untreated cells); ^*^ *p* < 0.05, ^**^ *p* < 0.01 vs. the RANKL only treatment group.

To confirm the viability of RAW 264.7 cells, we performed a CCK-8 assay. RAW 264.7 cells were treated with SR (125, 250, 500, 1,000 μg/ml) for 24 h, and the viability of RAW 264.7 cells was not affected after SR treatment. Then, to verify the cell viability in osteoclasts, we performed a CCK-8 assay. RAW 264.7 cells were treated with RANKL (100 ng/ml) and SR (125, 250, 500, 1,000 μg/ml) for 5 days, and the viability of osteoclasts was not affected after SR treatment. These results indicate that the osteoclast inhibitory effect of SR is not due to toxicity (Figures 1A,B). We conducted osteoclast experiments, as shown in Figure 1C. To examine the effect of SR on osteoclast differentiation in RAW 264.7 cells, we conducted TRAP staining and TRAP activity assays. As shown in Figures 1D,E, when only RANKL was added to RAW 264.7 cells, the number of multinucleated TRAP-positive cells stained red was increased. However, in the SR-treated cells, the number of multinucleated TRAP-positive cells stained red was decreased in a dose-dependent manner. In addition, TRAP activity in the differentiation medium was significantly decreased by SR treatment (Figure 1F).

### *Sparganii Rhizoma* Reduced the Pit Area for Bone Resorption of the F-Actin Ring for Osteoclast Cytoskeletal Structure

**FIGURE 2:**
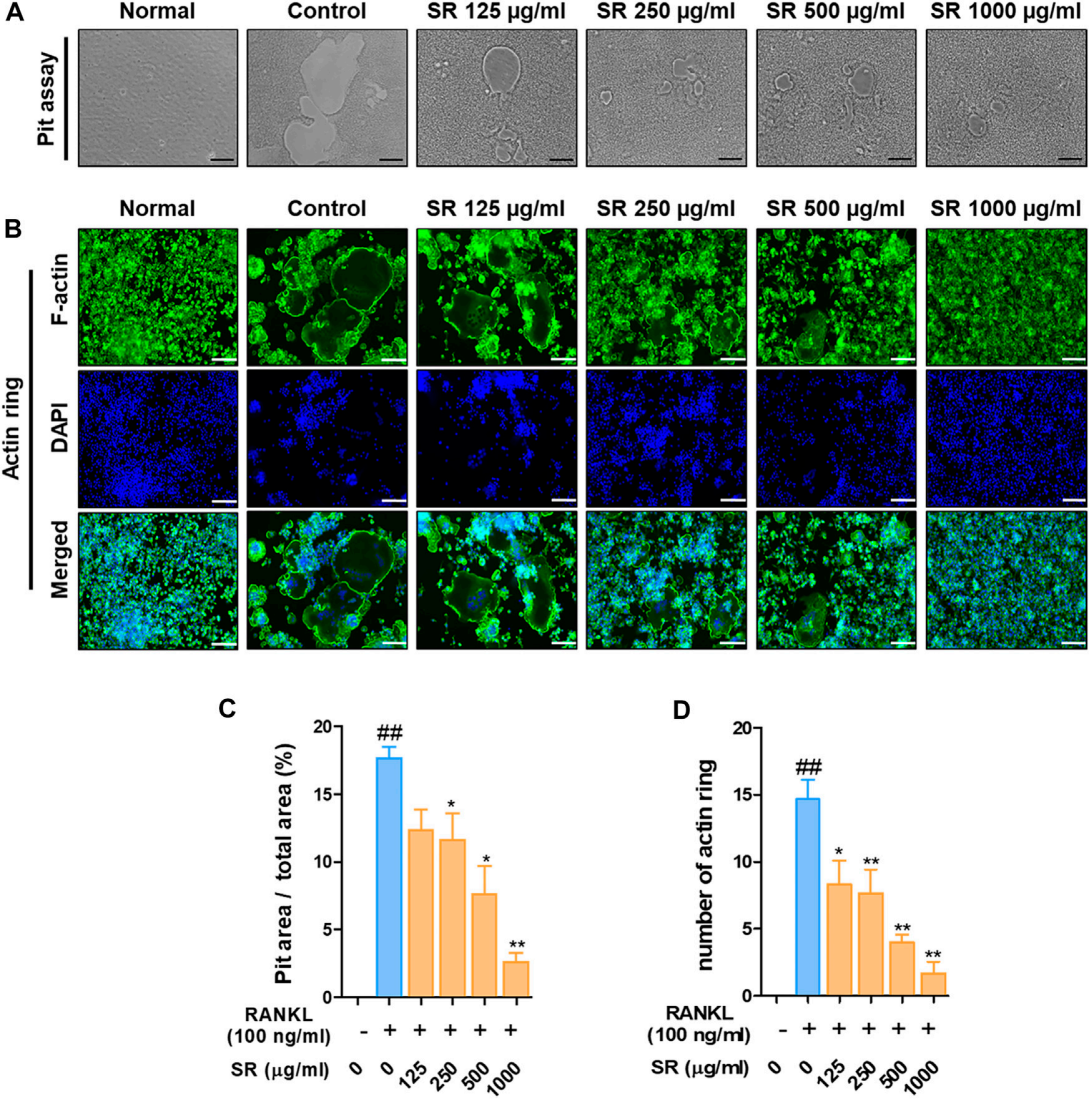
The effects of SR on bone resorption and F-actin ring formation in RANKL-induced RAW 264.7 cells. **(A)** Pit formation was captured using an inverted microscope (magnification, ×100; scale bar, 200 µm). **(B)** F-actin rings were stained with fluorescent phalloidin (magnification, ×100; scale bar, 200 µm). **(C)** The pit area was measured using ImageJ software. Data are presented as the mean ± SD (standard error of the mean) of three independent experiments. **(D)** The number of F-actin rings was counted. Data are presented as the mean ± SD of three independent experiments. Statistical analysis was performed using one-way ANOVA followed by Dunnett’s post hoc test. ^##^ *p* < 0.01 vs. the normal group (untreated cells); ^*^ *p* < 0.05, ^**^ *p* < 0.01 vs. the RANKL only treatment group.

To determine the effect of SR on bone resorption, we assessed the pit formation. As shown in Figure 2A, when only RANKL was added to RAW 264.7 cells, the pit area was increased compared with that of the nontreated cells. However, in the SR-treated cells, the pit area was considerably decreased in a dose-dependent manner. Afterward, we examined F-actin ring formation. The F-actin ring was considerably increased in the RANKL-treated cells compared with the nontreated cells, and the F-actin ring was decreased in the SR-treated cells (Figure 2B). Consistent with these results, the SR-treated cells had a significantly decreased pit area and number of F-actin rings. SR was shown to have a significant inhibitory effect on the bone resorption and F-actin ring formation (Figures 2C,D).

### *Sparganii Rhizoma* Decreased the Expression of TRAF6/NF-κB/MAPK Signaling Pathway

**FIGURE 3:**
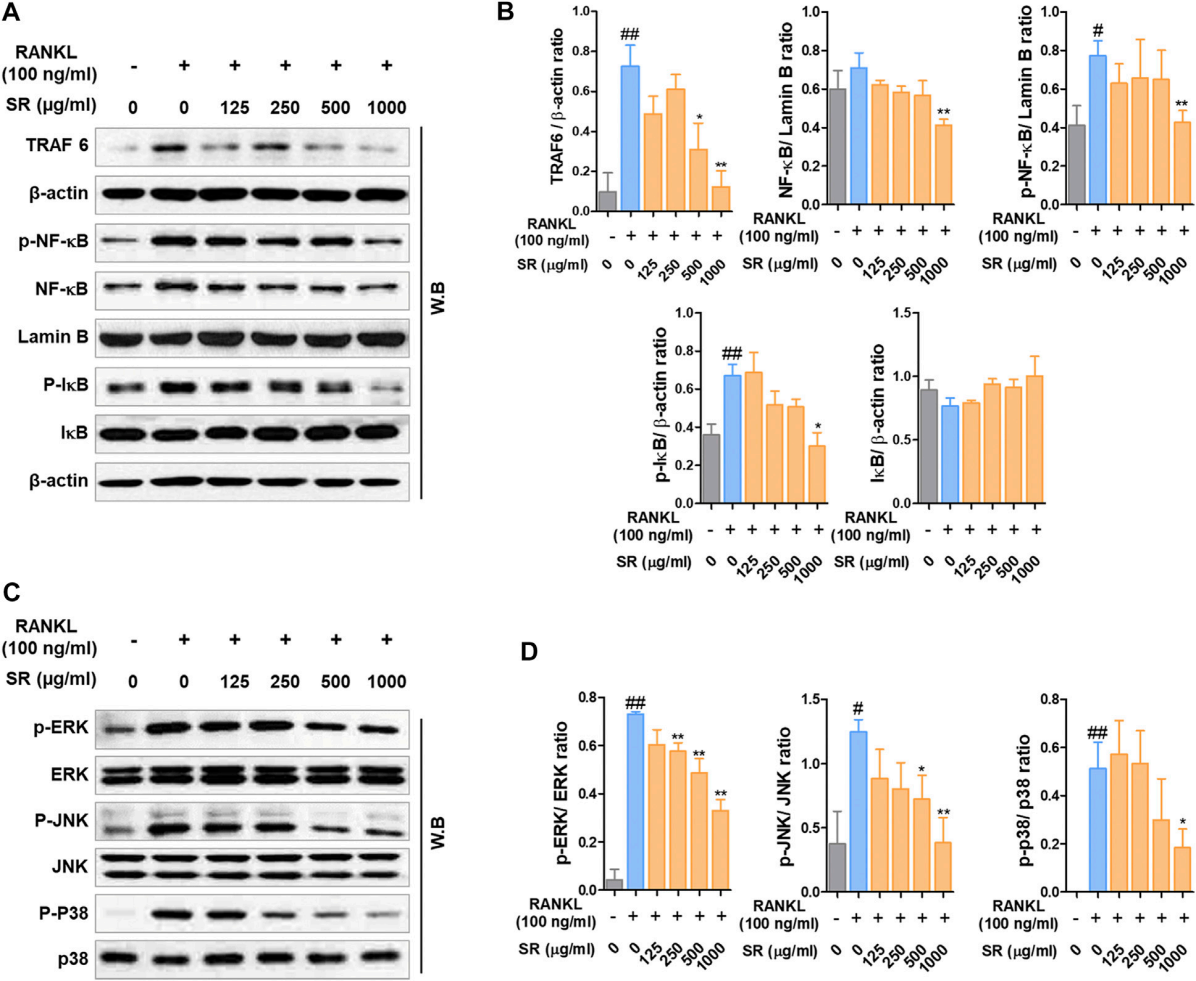
The effects of SR on the expression of TRAF6, NF-κB and MAPK signaling pathway. **(A)** Protein expression levels of TRAF6, p-NF-κB, NF-κB, p-IκB and IκB were measured by western blotting. **(B)** TRAF6, p-IκB and IκB were normalized to that of β-actin, which was used as a loading control. p-NF-κB and NF-κB were normalized to that of Lamin B, which was used as a loading control. **(C)** Protein expression levels of p-ERK, p-JNK, p-p38 were measured by western blotting. The bands were normalized to that of ERK, JNK and p38. Data are presented as the mean ± SD of three independent experiments. Statistical analysis was performed using one-way ANOVA followed by Dunnett’s post hoc test. ^#^ *p* < 0.05, ^##^ *p* < 0.01 vs. the normal group (untreated cells); ^*^ *p* < 0.05, ^**^ *p* < 0.01 vs. the RANKL only treatment group.

After demonstrating the inhibitory effect of SR on osteoclast differentiation, the role of SR on TRAF6/NF-κB signaling/MAPK signaling, an early transcription factor in osteoclast differentiation, was confirmed. As shown in Figures 3A,B, the expression of TRAF6 was significantly increased due to RANKL stimulation, and SR inhibited this increase. In addition, SR significantly inhibited nuclear translocation and phosphorylation of NF-κB and phosphorylation of IκB through RANKL stimulation and SR reduced the degradation of IκB induced by RANKL, but the difference was not significant. As presented in Figures 3C,D, RANKL stimulation significantly increased phosphorylation of ERK, JNK and p38, and SR significantly inhibited this increase.

### *Sparganii Rhizoma* Inhibited Transcription Factors Related to Osteoclast Differentiation

**FIGURE 4:**
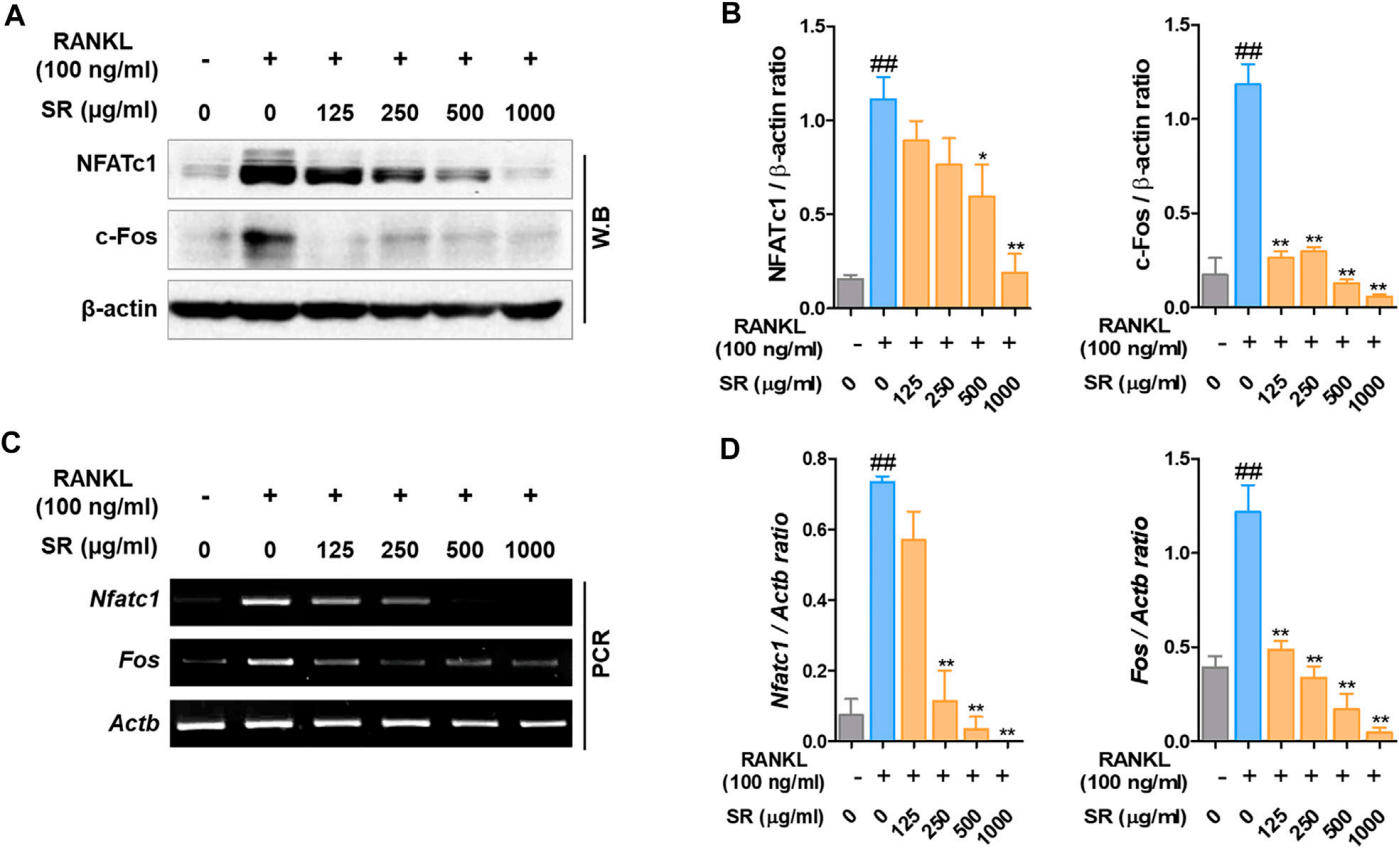
The effects of SR on NFATc1 and c-Fos levels in RAW 264.7 cells. **(A)** Protein expression levels of NFATc1 and c-Fos were measured by western blotting. **(B)** The bands were normalized to that of β-actin, which was used as a loading control. **(C)** mRNA expression levels in *Nfatc1* and *Fos* were measured by RT-PCR. The bands were normalized to that of β-actin (*Actb*). Data are presented as the mean ± SD of three independent experiments. Statistical analysis was performed using one-way ANOVA followed by Dunnett’s post hoc test. ^##^ *p* < 0.01 vs. the normal group (untreated cells); ^*^ *p* < 0.05, ^**^ *p* < 0.01 vs. the RANKL only treatment group.

To determine the expression of the transcription factors involved in the osteoclast differentiation effect of SR, we measured the expression levels of NFATc1 and c-Fos by western blots and RT-PCR. As shown in Figure 4A, after RANKL treatment, the protein expression of NFATc1 and c-Fos was increased; by comparison, the expression of NFATc1 and c-Fos was decreased by SR treatment. As a result of quantification using β-actin, the protein expression of NFATc1 and c-Fos was shown to be reduced upon SR treatment compared with RANKL treatment, consistent with the visual results (Figure 4B). In the RANKL treatment group, the mRNA expression of *Nfatc1* and *Fos* was increased compared to that in the nontreatment group. However, in the SR treatment group, the mRNA expression of *Nfatc1* and *Fos* declined dramatically compared to that in the RANKL treatment group (Figure 4C). As a result of quantification using *Actb*, the mRNA levels of *Nfatc1* and *Fos* were found to decrease upon SR treatment compared to those of the RANKL treatment group, consistent with the visual results (Figure 4D).

### *Sparganii Rhizoma* Suppressed the Expression of Osteoclast-Related Genes

**FIGURE 5:**
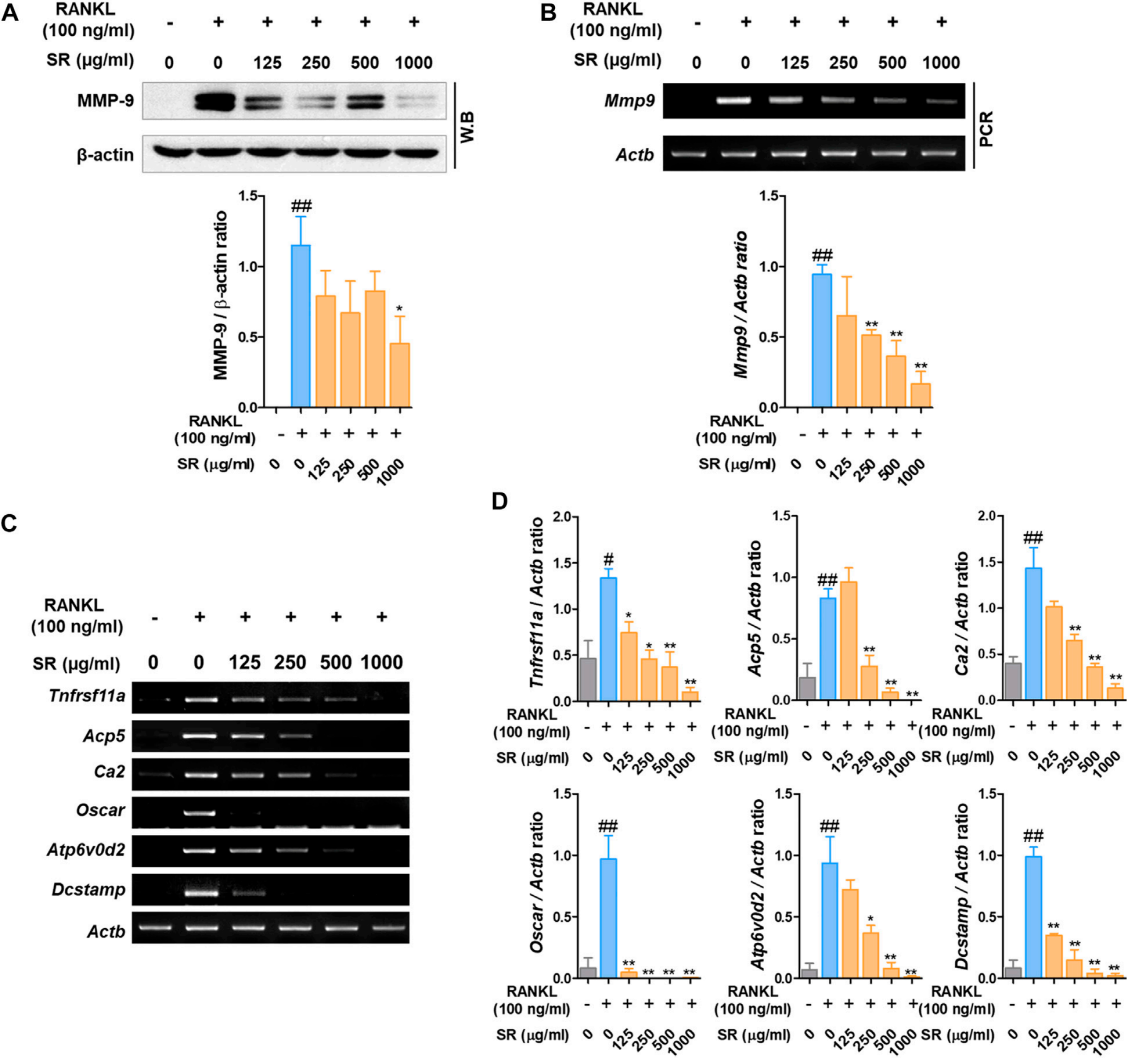
The effects of SR on the expression of osteoclast-related genes in RANKL-induced RAW 264.7 cells. **(A)** Protein expression of MMP-9 was determined using Western blotting and **(B)** mRNA expression of MMP-9 (*Mmp9*) was determined using RT-PCR. The bands were normalized to that of β-actin (*Actb*). **(C)** mRNA expression of RANK (*Tnfrsf11a*), TRAP (*Acp5*), CA2 (*Ca2*), OSCAR (*Oscar*), ATP6v0d2 (*Atp6vod2*) and DC-STAMP (*Dcstamp*) was determined using RT-PCR. **(D)** The bands were normalized to that of β-actin (*Actb*). Data are presented as the mean ± SD of three independent experiments. Statistical analysis was performed using one-way ANOVA followed by Dunnett’s post hoc test. ^#^ *p* < 0.05, ^##^ *p* < 0.01 vs. the normal group (untreated cells); ^*^ *p* < 0.05, ^**^ *p* < 0.01 vs. the RANKL only treatment group.

Matrix metalloproteinase-9 (MMP-9) is known as a factor related to osteoclast bone resorption. To determine the effect of SR on the expression of MMP-9, we performed Western blotting and RT-PCR. As shown in Figures 5A,B, after RANKL treatment, the protein and mRNA expression of MMP-9 was increased; by comparison, the expression of MMP-9 was decreased by SR treatment. To determine the effect of SR on the expression of osteoclast-related genes, which are bone resorption and osteoclast differentiation markers, we performed RT-PCR. As shown in Figure 5C, after RANKL treatment, the mRNA expression of osteoclast-related genes, such as RANK (*Tnfrsf11a*), TRAP (*Acp5*), carbonic anhydrase II (CA2/*Ca2*), osteoclast-associated receptor (OSCAR/*oscar*), ATPase H+ transporting v0 subunit d2 (ATP6v0d2/*Atp6v0d2*) and dendritic cell-specific transmembrane protein (DC-STAMP/*Dcstamp*), was increased, while the mRNA expression of osteoclast-related genes was considerably decreased by SR treatment. As a result of quantification using *Actb*, the mRNA levels were found to increase after RANKL treatment, and the expression of osteoclast-related genes was decreased by SR treatment (Figure 5D).

### *Sparganii Rhizoma* Significantly Increased Osteoblast Differentiation in MC3T3-E1 Cells

**FIGURE 6:**
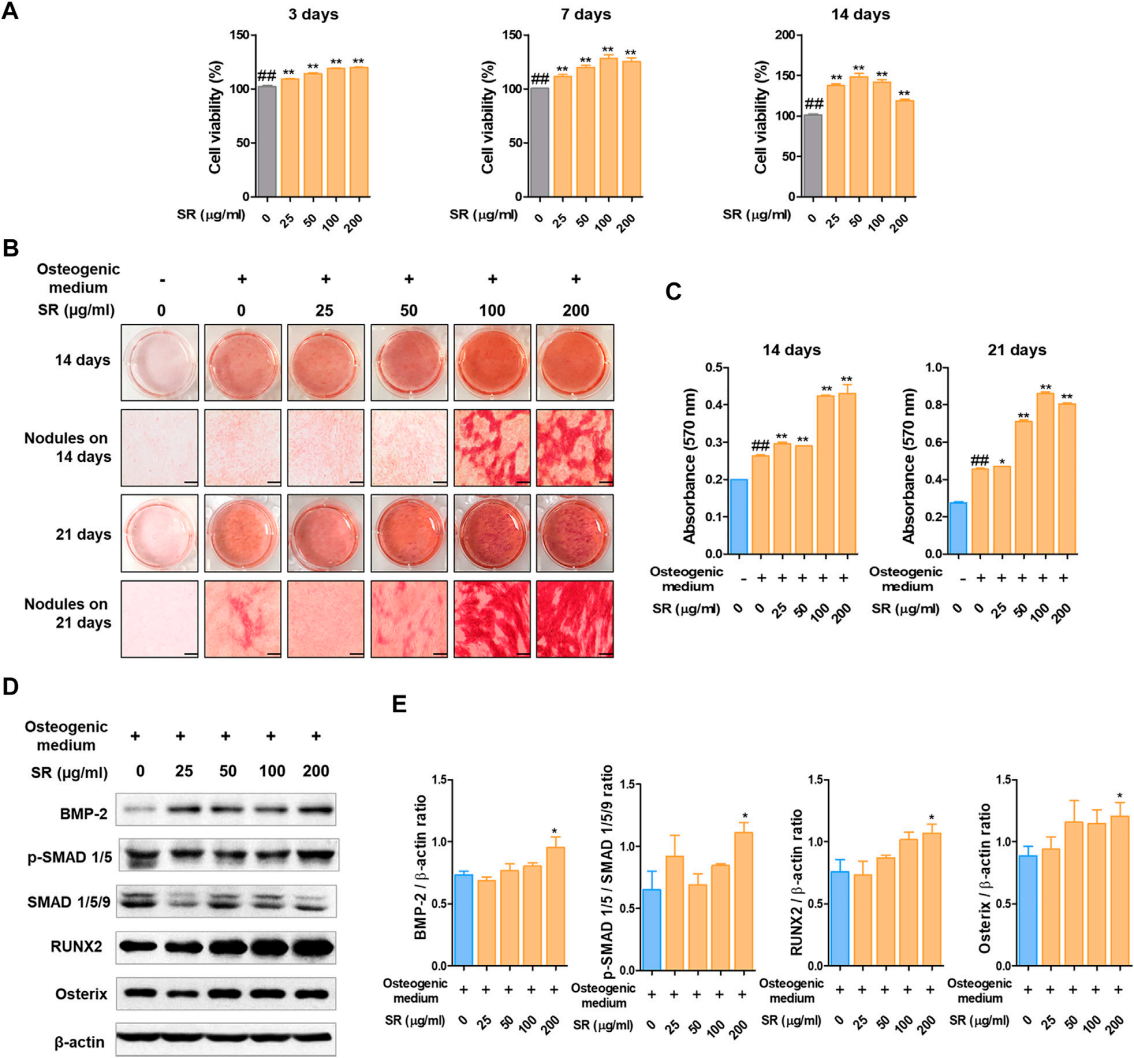
The effects of SR on osteoblast differentiation. **(A)** MC3T3-E1 cell viability was analyzed by CCK-8 assays at 3, 7 and 14 days. **(B)** Calcified nodules produced by osteoblasts were stained with Alizarin Red S for 14 and 21 days. **(C)** The absorbance of Alizarin Red S staining dye was measured using an ELISA reader at 450 nm. **(D)** Protein expression levels of BMP-2, p-Smad 1/5, RUNX-2 and Osterix were measured by western blotting. **(E)** BMP-2, RUNX-2 and Osterix levels were normalized to that of β-actin. p-Smad 1/5 levels was normalized to that of Smad 1/5/9. Data are presented as the mean ± SD of three independent experiments. Statistical analysis was performed using one-way ANOVA followed by Dunnett’s post hoc test. ^##^ *p* < 0.01 vs. the normal group (untreated cells); ^*^ *p* < 0.05, ^**^ *p* < 0.01 vs. the osteogenic medium-treated cells.

To investigate the underlying mechanisms by which SR increases osteoblast differentiation, we examined the effects of SR in MC3T3-E1 cells. In this study, we initially examined the effect of SR treatment on MC3T3-E1 cell viability. The viability of MC3T3-E1 cells was not affected by treatment with SR for 3, 7 or 14 days (Figure 6A). Following 14 days of osteoblast differentiation, calcified nodules were formed at an earlier stage in the SR treatment group than in the osteogenic medium treatment group. Following 21 days of osteoblast differentiation, calcified nodules were increased by treatment with osteogenic medium compared with those of the growth medium treatment group. In addition, SR at concentrations of 100 and 200 μg/ml resulted in significantly increased calcified nodules compared with those of the osteogenic medium treatment (Figure 6B). Moreover, the absorbance of Alizarin Red S staining dye was significantly increased by SR treatment in a dose-dependent manner after 14 and 21 days compared with that of the osteogenic medium treatment group (Figure 6C). Afterward, the effect of SR on the osteoblast differentiation pathway at the protein level was verified. Therefore, as shown in Figures 6D,E, SR upregulated the protein expression of BMP-2, RUNX2 and Osterix and p-SMAD 1/5.

### Change in Body and Uterine Weight and Serum Levels of TRAP, ALP, AST, and ALT

**FIGURE 7:**
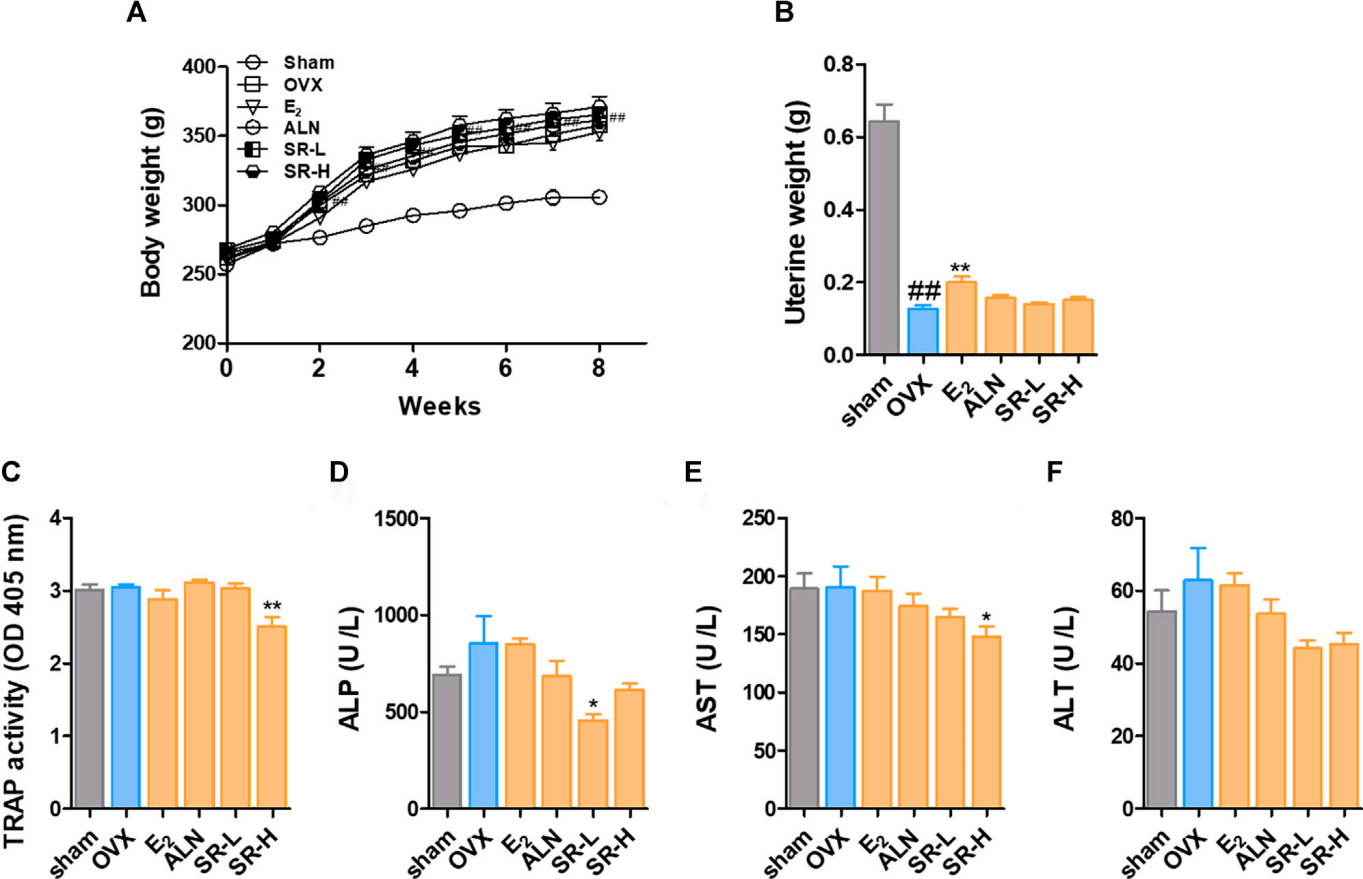
Change in body weight and serum levels of TRAP, ALP, AST and ALT in the OVX model. **(A)** Body weight was measured once a week for 8 weeks. **(B)** The uterine weight was measured after sacrifice. The serum levels of **(C)** TRAP, **(D)** ALP, **(E)** AST, and **(F)** ALT were measured using an ELISA reader. The results are presented as the mean ± SEM of each experimental group (*n* = 8). Statistical analysis was performed using one-way ANOVA followed by Dunnett’s post hoc test. ^#^ *p* < 0.05, ^##^ *p* < 0.01 vs. the normal group (sham-operation group); ^*^ *p* < 0.05, ^**^ *p* < 0.01 vs. the control group (OVX-induced group).

To investigate the effect of SR on the OVX-induced model, we orally administered SR to rats for 8 weeks. After 2 weeks, the OVX-induced group had an increased body weight compared with the sham group. In addition, the weight was not significantly different in the E_2_, ALN, SR-L and SR-H groups compared to the OVX group (Figure 7A). As shown in Figure 7B, uterine weight was significantly reduced in the OVX group compared with the sham group. The E_2_ group had decreased uterine weight, but the ALN, SR-L and SR-H groups did not show changes. Next, to determine the effect of SR on the levels of TRAP, ALP, AST, and ALT in serum, we performed ELISAs. After 8 weeks, the levels of TRAP were significantly decreased in the SR-H group. However, TRAP activity was unchanged in the E_2_, ALN and SR-L groups (Figure 7C). The ALP levels were increased in the OVX-induced group compared with the sham group. The ALP levels were significantly decreased in the SR-L group and not significantly decreased in the E_2_, ALN, and SR-H groups, but the ALP levels were decreased compared to those in the sham group (Figure 7D). To investigate hepatotoxicity by SR treatment, we analyzed AST and ALT as indicators of hepatotoxicity. The E_2_, ALN and SR-L groups did not show hepatotoxicity compared with the OVX group, and the SR-H group had decreased levels of AST. In addition, the levels of ALT did not affect the E_2_, ALN and SR-H groups. However, the levels of ALT were reduced in the SR-L group (Figures 7E,F).

### *Sparganii Rhizoma* Prevents Bone Loss in the OVX-Induced Model

**FIGURE 8:**
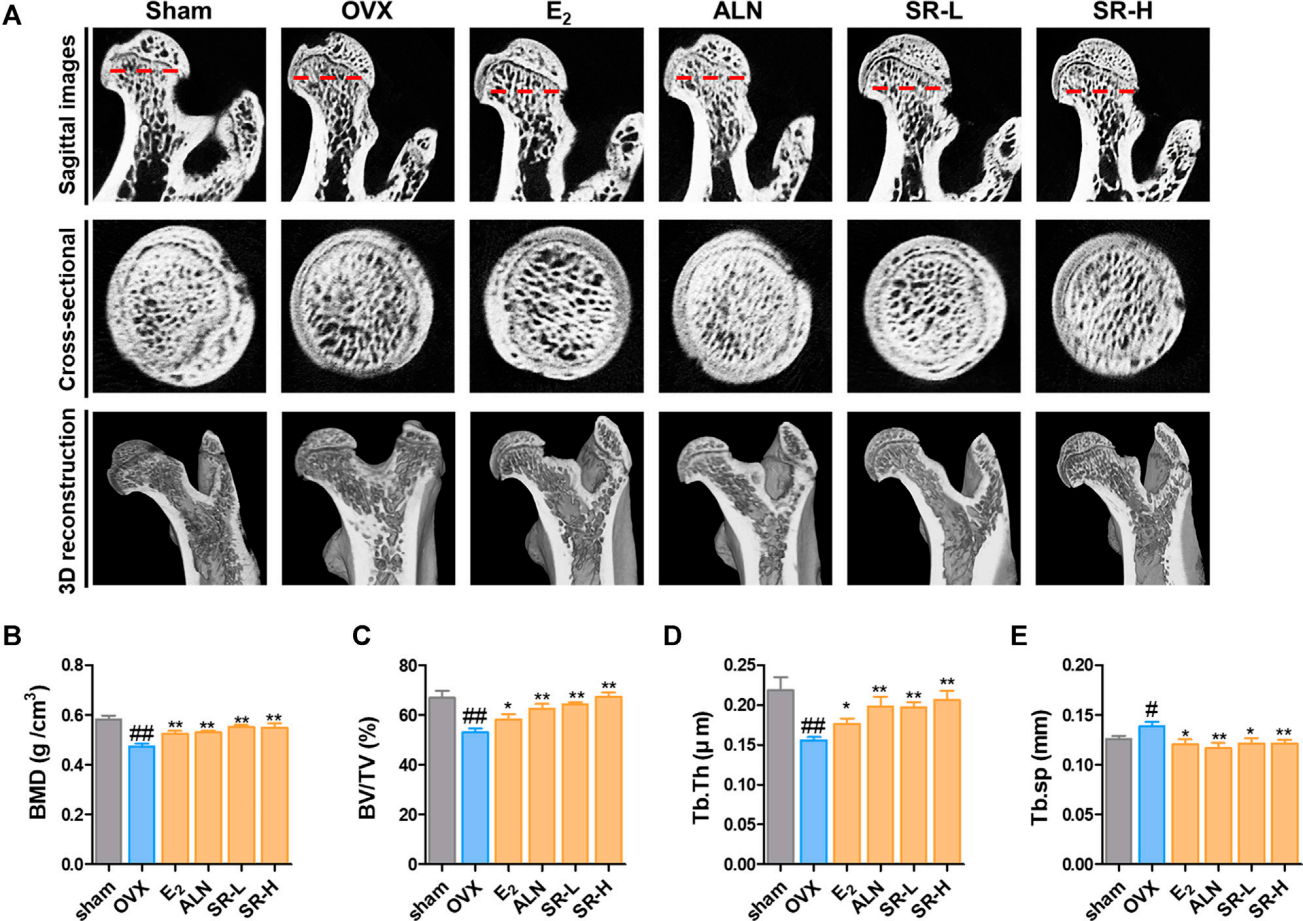
Effects of SR on bone loss in the OVX-induced model. **(A)** Micro-CT analysis of the femurs. **(B)** BMD, **(C)** BV/TV, **(D)** Tb.Th and **(E)** Tb. sp of femurs were measured using micro-CT. The results are presented as the mean ± SEM of each experimental group (*n* = 8). Statistical analysis was performed using one-way ANOVA followed by Dunnett’s post hoc test. ^#^ *p* < 0.05, ^##^ *p* < 0.01 vs. the normal group (sham-operation group); ^*^ *p* < 0.05, ^**^ *p* < 0.01 vs. the control group (OVX-induced group).

To confirm the change in bone metabolism in the OVX-induced model, we examined the femur using micro-CT. In the sagittal images, bone loss increased in the OVX group compared to the sham group. However, bone loss was decreased in the E_2_, ALN, SR-L, and SR-H groups. The same result was found in the cross-sectional and 3D reconstruction images (Figure 8A). Next, we explored the effect of SR in bone microarchitecture. BMD, BV/TV and Tb.Th were significantly decreased in the OVX group compared with the sham group. The E_2_, ALN, and SR groups had considerably increased BMD, BV/TV and Tb.Th values compared to the OVX group (Figures 8B–D). Tb. sp was significantly increased in the OVX group compared to the sham group, and the E_2_, ALN and SR groups had decreased Tb. sp values compared to the OVX group (Figure 8E).

### *Sparganii Rhizoma* Suppressed Trabecular Bone Loss and Osteoclast Formation and Increased Osteoblast Differentiation

**FIGURE 9:**
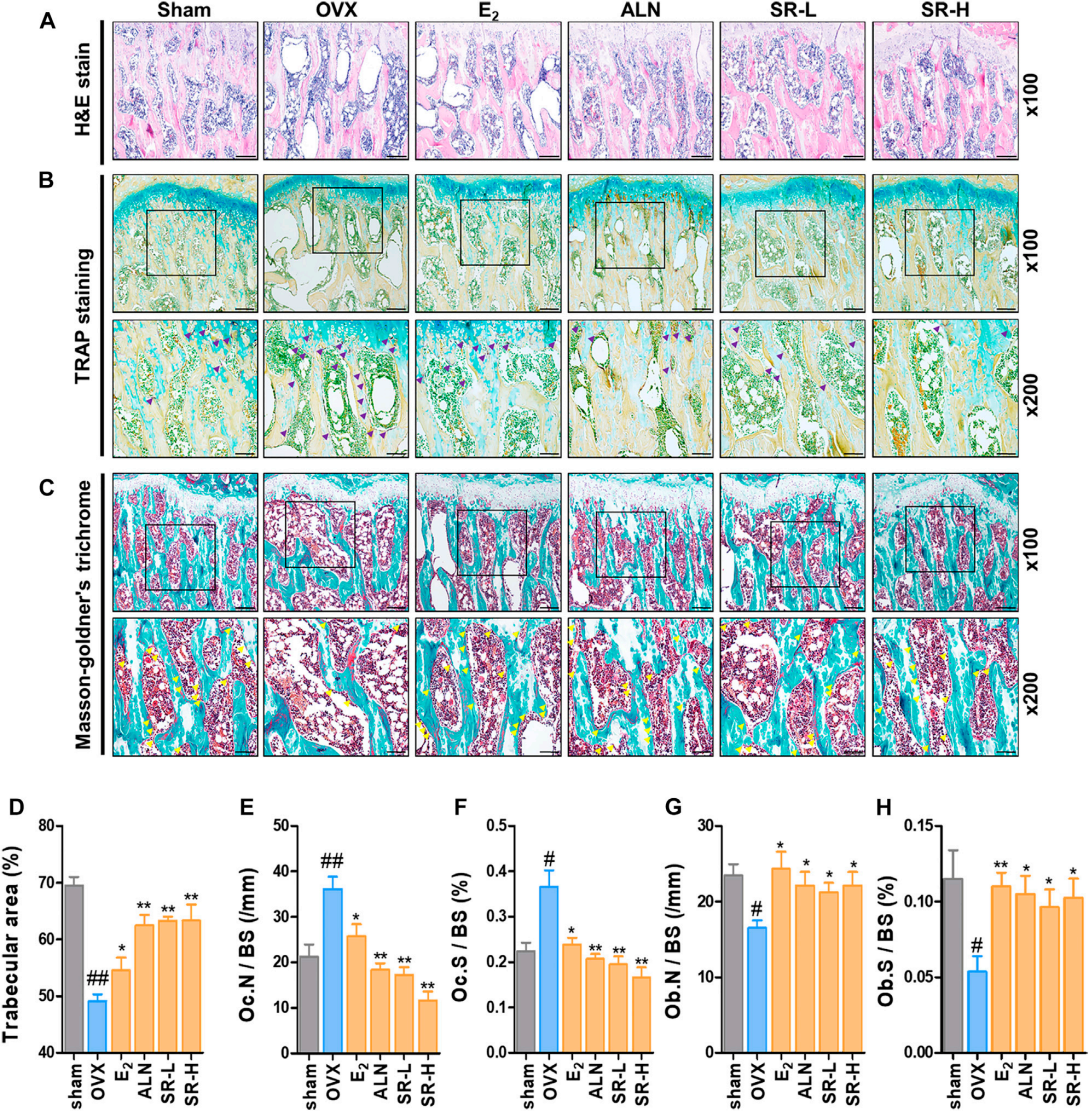
Effect of SR on trabecular area, osteoclasts and osteoblast in the OVX-induced rats. **(A)** The trabecular area was measured using H&E staining of femur tissue. **(B)** The number of osteoclasts (purple) in the femoral tissue was verified through TRAP staining. **(C)** The number of osteoblasts (yellow) in the femoral tissue was measured through masson-goldner’s trichrome staining. **(D)** Trabecular area, **(E)** Oc.N /BS (/mm). **(F)** Oc.S /BS (%). **(G)** Ob. N/BS and **(F)** Ob. S/BS were measured using ImageJ version 1.46. The results are presented as the mean ± SEM of each experimental group (*n* = 8). Statistical analysis was performed using one-way ANOVA followed by Dunnett’s post hoc test. ^##^ *p* < 0.01, ^#^ *p* < 0.05 vs. the normal group (sham-operation group); ^*^ *p* < 0.05, ^**^ *p* < 0.01 vs. the control group (OVX-induced group).

To further verify the effect of SR on OVX-induced bone loss, we used histological staining by H&E staining. The results showed that trabecular bone loss was significantly increased in the OVX group compared with the sham group. However, trabecular bone loss was decreased in the E_2_, ALN and SR groups compared with the OVX group (Figures 9A,D). We performed TRAP staining and masson-goldner’s trichrome staining to measure the expression of osteoclasts and osteoblasts in the femoral tissue, respectively. In addition, the surface and number of TRAP-positive cells in the OVX group increased compared to the sham group, and the surface and number of TRAP-positive cells in the E_2_, ALN and SR groups decreased compared to the OVX group (Figures 9B,E,F). Also, the surface and number of osteoblasts was decreased in the OVX group compared to the sham group, and the surface and number of osteoblasts was increased in the E_2_, ALN and SR groups compared to the OVX group (Figures 9C,G,H).

### *Sparganii Rhizoma* Decreased the Number of CTK- and NFATc1-Positive Cells and Increased the Number of BMP2-Positive Cells

**FIGURE 10:**
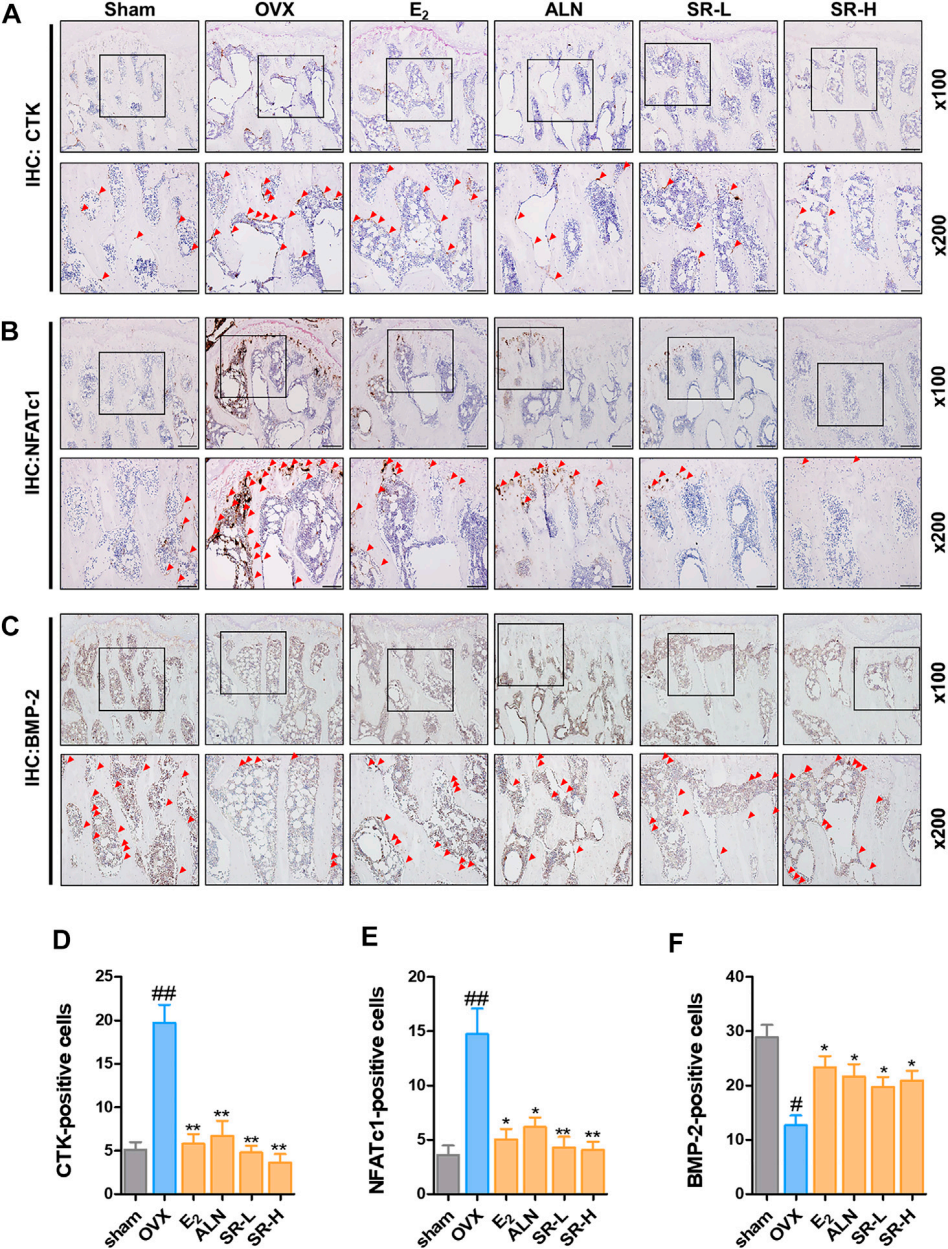
Effect of SR on histopathological examination in the OVX-induced rats. **(A)** CTK-, **(B)** NFATc1- and **(C)** BMP-2-positive cells were measured using IHC staining. **(D)** The number of CTK- **(E)** NFATc1- and **(F)** BMP-2-positive cells was counted using ImageJ version 1.46. The results are presented as the mean ± SEM of each experimental group (*n* = 8). Statistical analysis was performed using one-way ANOVA followed by Dunnett’s post hoc test. ^##^ *p* < 0.01, ^#^ *p* < 0.05 vs. the normal group (sham-operation group); ^*^ *p* < 0.05, ^**^ *p* < 0.01 vs. the control group (OVX-induced group).

To determine the effect of SR on the number of CTK, NFATc1 and BMP-2 cells, we performed IHC staining. The number of CTK- and NFATc1-positive cells in the OVX group was notably higher than that in the sham group (Figures 10A,B). However, the number of CTK- and NFATc1-positive cells was considerably reduced in the E_2_, ALN and SR groups (Figures 10D,E). The number of BMP-2-positive cells in the OVX group was significantly decreased than that in the sham group. However, the number of BMP-2-positive cells was significantly increased in the E_2_, ALN and SR groups (Figures 10C,F).

### Identification of Kaempferol as an *Sparganii Rhizoma* Component

**FIGURE 11:**
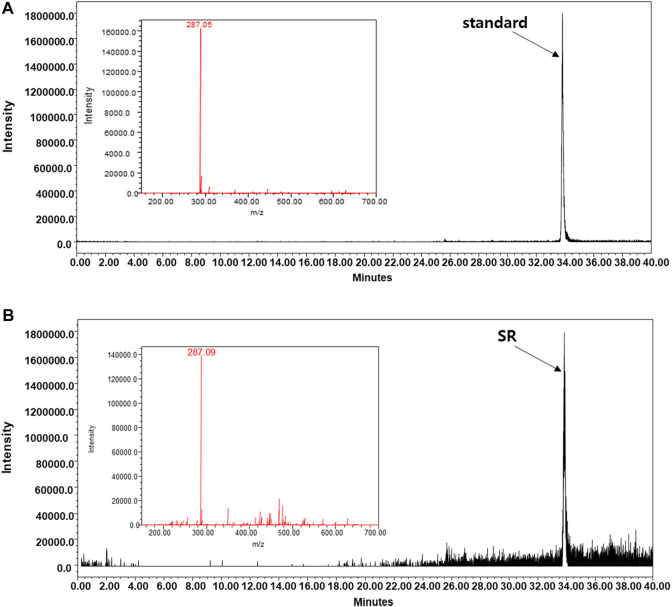
LC-MS analysis was carried out using the A Waters e2695 system. Kaempferol was confirmed in the **(A)** standard and **(B)** SR extracts.

To determine the quality of SR, we conducted LC/MS analysis by measuring the content of a well-known compound of SR, kaempferol. Similar to the peaks of kaempferol, SR extract samples were detected at the same retention times, and kaempferol was identified as a component in the SR extract (Figures 11A,B).

### Comparison of Effects of *Sparganii Rhizoma* and Kaempferol on Osteoclasts and Osteoblasts

**FIGURE 12:**
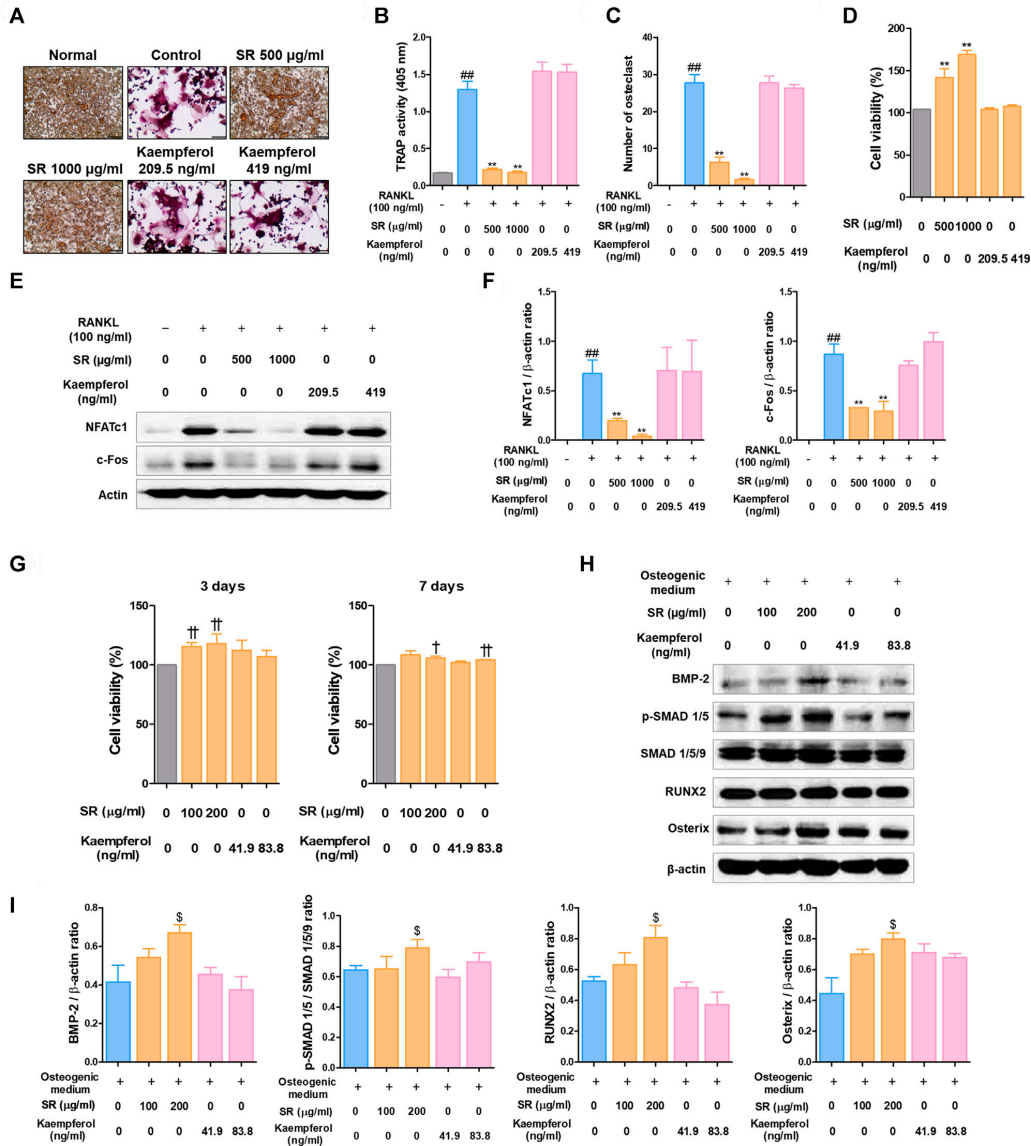
Comparative analysis of pharmacological effects of SR and kaempferol on the differentiation of osteoclasts and osteoblasts. **(A)** TRAP-positive cells were stained using a TRAP kit. **(B)** TRAP activity was measured using an ELISA reader. **(C)** A number of TRAP-positive cells with >3 nuclei were counted using an inverted microscope (magnification, ×100). **(D)** RAW 264.7 cell viability was measured using a CCK-8 assay kit. **(E)** Protein expression levels of NFATc1 and c-Fos were measured by western blotting. **(F)** The bands were normalized to that of β-actin, which was used as a loading control. **(G)** MC3T3-E1 cell viability was analyzed by CCK-8 assays at 3 and 7 days. **(H)** Protein expression levels of BMP-2, p-Smad 1/5, RUNX-2 and Osterix were measured by western blotting. **(I)** BMP-2, RUNX-2 and Osterix levels were normalized to that of β-actin. p-Smad 1/5 levels was normalized to that of Smad 1/5/9. Data are presented as the mean ± SD of three independent experiments. Statistical analysis was performed using one-way ANOVA followed by Dunnett’s post hoc test. ^##^ *p* < 0.01 vs. the normal group (untreated cells, RAW 264.7 cells); ^*^ *p* < 0.05, ^**^ *p* < 0.01 vs. the RANKL only treatment group. ^†^ *p* < 0.05, ^††^ *p* < 0.01 vs. the normal group (untreated cells, MC3T3-E1 cells); ^$^ *p* < 0.05 vs. the osteogenic medium-treated cells.

The positive results of kaempferol on osteoclasts and osteoblasts have been reported in various previous studies (Guo et al., 2012; Kim et al., 2018). In order to confirm whether the pharmacological effect of SR is dependent on kaempferol, the main component, we conducted osteoclast and osteoclast experiments based on the concentration of kaempferol contained in SR. Based on the LS-MS data, the content of kaempferol in 1 g of SR was 0.419 mg. Therefore, the amount of kaempferol contained in 1,000 μg/ml SR is 419 ng/ml, and the amount of kaempferol contained in 200 μg/ml SR is 83.8 ng/ml. As shown in Figures 12A–C, SR inhibited the differentiation and activity of osteoclasts as in the previous experimental results, but kaempferol had no effect on the number of osteoclasts and TRAP activity in the medium. The concentrations of SR and Kaempferol in the osteoclast experimental did not show cytotoxicity (Figure 12D). Protein expression of NFATc1/c-Fos also strongly inhibited SR, but kaempferol had no significant effect (Figures 12E,F). The concentrations of SR and kaempferol in the osteoblast experimental did not show cytotoxicity (Figure 12G). SR showed a significant increase in the expression of BMP-2, p-SMAD1/5, RUNX2 and Osterix, which are key factors for osteoblast differentiation, but kaempferol did not show a significant effect on the expression of these proteins. These results indicate that the concentration of kaempferol contained in SR does not have a significant effect on osteoclasts and osteoblasts. In addition, it means that the pharmacological effect of SR is not mediated by kaempferol, but is a comprehensive effect of various components constituting SR. Therefore, this study is considered to be different from the existing studies on kaempferol.

## Discussion

**FIGURE 13:**
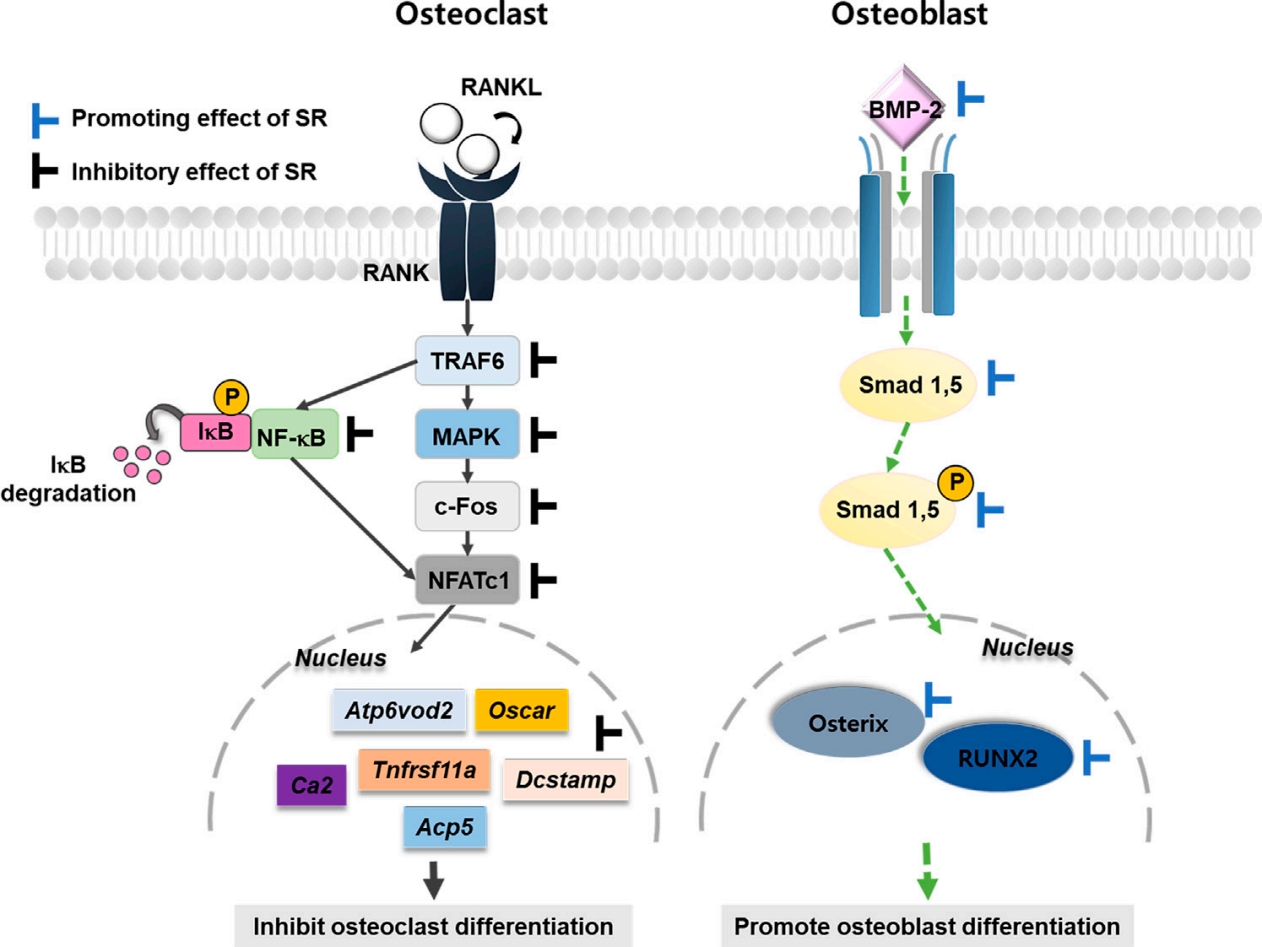
Schematic diagram of SR in bone metabolism.

Osteoporosis is caused by an imbalance in bone remodeling. Therefore, it is important to inhibit the activity of osteoclasts or increase the activation of the differentiation of osteoblasts (Feng and McDonald, 2011). This study used two cell lines to evaluate the effects of SR on osteoclasts and osteoblasts. RAW 264.7 cells are derived from mouse monocytes/macrophages and are suitable for establishing an osteoclast differentiation model. MC3T3-E1 cells are osteoblast precursor cell lines derived from mouse calvaria. Experimental results *in vitro* demonstrated that SR inhibited osteoclast differentiation via the NFATc1/c-Fos signaling pathway. In addition, SR increased osteoblast differentiation via an upregulated BMP-2/RUNX-2 signaling pathway. *In vivo*, SR also inhibited bone loss in the OVX-induced model (Figure 13).

RAW 264.7 cells originate from hematopoietic precursors of the monocyte/macrophage lineage (Teitelbaum and Ross, 2003; Hartley et al., 2008). RAW 264.7 cells are known to be suitable for *in vitro* studies of osteoclast formation and function (Teitelbaum and Ross, 2003). TRAP is an iron-containing enzyme common to bones and the immune system. In addition, TRAP has been used as a representative histochemical marker for osteoclasts (Ballanti et al., 1997; Boyle et al., 2003). Pit formation assays are commonly used to examine osteoclast differentiation and bone resorption ability (Marchisio et al., 1984; Bradley and Oursler, 2008). In the present study, we demonstrated that SR significantly decreased the number of osteoclasts and activity and significantly reduced the pit area. Osteoclasts induce skeletal formation during the bone resorption process and attach to the bone. Additionally, osteoclast actin is organized into one large ring to separate the extracellular space. The formation of the actin ring is an important marker for the bone resorption of osteoclasts (Marchisio et al., 1984; Matsubara et al., 2017). The results of the current study showed that SR significantly decreased F-actin formation in RANKL-induced osteoclasts. Taken together, these results suggest that SR suppresses osteoclast differentiation, formation and resorption.

RANK, like many TNFR family proteins, transduces biochemical signals following recruitment of intracellular adapter TNF receptor-associated factor (TRAF) proteins, and TRAF6 plays an essential role in RANKL-mediated NF-κB activation (Armstrong et al., 2002). Previous studies have shown that TRAF6 deficiency inhibits RANKL-induced JNK and p38 activation (Lomaga et al., 1999). NF-κB translocates to the nucleus via RANKL-RANK binding and plays an important role in the early stage of osteoclast differentiation (Grande et al., 2015). Previous studies have shown that mice deficient in NF-κB1 and NF-κB2 cannot differentiate into osteoclasts, leading to osteopetrosis (Iotsova et al., 1997). The MAPK signaling pathway is known to play an important role in osteoclast differentiation. ERK is associated with osteoclast survival, and JNK and p38 promote osteoclast differentiation (Matsumoto et al., 2000; Miyazaki et al., 2000). In this study, SR treatment reduced phosphorylation of IκB and NF-κB. In addition, SR treatment inhibited the MAPK signaling pathway by reducing phosphorylation of ERK, JNK and p38. These results suggest that SR can regulate osteoclast differentiation by inhibiting phosphorylation of NF-κB and MAPK.

Activation of the TRAF6, NF-κB and MAPK signaling pathways by the interaction of RANKL and RANK promotes the activation of several downstream transcription factors, such as NFATc1 and c-Fos (Wada et al., 2006). In previous studies, overexpression of NFATc1 accelerated osteoclast differentiation and increased osteoclast formation independent of RANKL. NFATc1-deficient embryonic stem cells could not differentiate into osteoclasts, even in the presence of RANKL (Takayanagi et al., 2002; Kim and Kim, 2014). In addition, c-Fos deficiency causes severe osteoporosis due to a lack of osteoclasts (Arai et al., 2012). In this study, SR significantly inhibited the protein and mRNA expression of NFATc1 and c-Fos. These results indicate that SR can reduce osteoclast formation, differentiation and functions by inhibiting the NFATc1 and c-Fos signaling pathways.

RANK (*Tnfrsf11a*) is expressed on the surface of mature T cells and hematopoietic precursors. Activation of the *Tnfrsf11a* receptor triggers intracellular signaling mediated by the interaction of intracellular I, II and III domains, adapter proteins, and TNF receptor-related factors (TRAFs). RANK-deficient mice were characterized by severe osteopetrosis due to blockade of osteoclast differentiation (Wong et al., 1997; Dougall et al., 1999). c-Fos and NFATc1 upregulate the expression of osteoclast-specific genes such as TRAP (*Acp5*)*,* MMP-9 (*Mmp9*)*,* CA2 (*ca2*)*,* OCSAR (*Oscar*)*,* ATP6vod2 (*Atp6v0d2*)*,* and DC-STAMP (*Dcstamp*)*. Acp5, Mmp9* and *Ca2* are involved in osteoclast differentiation, which promotes bone resorption (Zhao et al., 2010). Additionally, *Mmp9* has been shown to be important for bone development and repair (Colnot et al., 2003). *Ca2* affects the acidity of the bone surface, and its expression is upregulated by c-Fos signaling. When *CA2* is deficient, it has been found that nonfunctional osteoclasts cause osteoporosis (Sly et al., 1983; David et al., 2001). OSCAR is expressed in monocytes, macrophages, dendritic cells and osteoclasts and is a collagen-activating receptor that stimulates osteoclast differentiation. In a previous study, in the case of mononuclear cells from patients with high *Oscar* expression, the potential to differentiate into osteoclasts was improved compared to that of cells with low expression (Herman et al., 2008). Thus, *Oscar* is an important factor in osteoclast formation. *Atp6vod2* and *Dcstamp* are essential factors for cell-cell fusion (Kim et al., 2008). According to previous studies, *Atp6vod2*-deficient mice developed osteofossilosis due to abnormal osteoclast maturation (Lee et al., 2006). The osteoclasts isolated from *Dcstamp* knockout mice were osteoclasts with a single nucleus due to the lack of cell-cell fusion. In our study, SR inhibited the expression of osteoclast-specific genes (Yagi et al., 2005). These results demonstrated that SR suppressed osteoclast-specific genes through inhibition of c-Fos and NFATc1.

MC3T3-E1 cells were established from C57BL/6 mouse calvaria and are known to be suitable for *in vitro* experiments of osteoblast differentiation (Quarles et al., 1992). Alizarin Red S staining and Von Kossa staining are methods for staining calcium in mineralized nodule substrates. Calcified nodules are indicators of osteoblast differentiation (Puchtler et al., 1969; Bills et al., 1971). In this study, mineralized nodules increased in the osteogenic medium group compared with the growth medium group. SR significantly increased the calcified nodules in a concentration-dependent manner. These results indicate that SR plays an important role in promoting mineralization in MC3T3-E1 cells. The differentiation and maturation of osteoblasts is regulated by several factors. BMP-2 and RUNX-2 are key factors that induce bone development and promote osteoblastic differentiation and bone formation. BMP-2 binds to receptors, resulting in the phosphorylation of SMAD, which then moves to the nucleus. Activated SAMD is translocated into the nucleus, and these signaling pathways activate RUNX-2. With increased RUNX2 activation, ALP (*Alpl*), COL1 (*COL1a1*) and BSP (*Ibsp*) are induced. Runx2 is an essential and important factor for osteoblast differentiation and chondrocyte maturation (Hay et al., 2001; Komori, 2010). Runx2-deficient mice lack osteoblasts and show little expression of bone matrix protein genes (Komori et al., 1997). Osterix is an osteoblast-specific transcription factor that activates a gene repertoire during osteoblast differentiation into mature osteoblasts and osteocytes. Osterix mutant embryos do not form bones and do not express osteoblast-specific marker genes (Cao et al., 2005; Sinha and Zhou, 2013). The results of the present study indicated that SR upregulates the expression of BMP-2, RUNX2 and Osterix and p-SAMD 1/5.

The OVX-induced rat model is mainly used in postmenopausal osteoporosis research and shares clinical characteristics with human osteoporosis. In a previous study, the OVX model showed an increase in body weight and atrophy of the uterus due to estrogen deficiency (Kalu, 1991; Eastell et al., 2016). In this study, the weight of the OVX group increased significantly from 2 weeks. In addition, uterine weight was significantly reduced in the OVX group compared to the sham group. These results indicate that the OVX model was successfully established. However, SR did not affect body or uterine weight. The results suggest that SR did not show an estrogen-like effect. Osteoclasts increase the levels of TRAP in the serum (Wronski et al., 1988). In this study, serum TRAP levels were increased in the OVX group compared to the sham group, but the difference was not significant. The reason for this small difference is not known, but it is assumed that this is a problem caused by the short experimental period. The ALP isoenzyme is derived from the bone and liver. ALP is a marker of osteoblast activity and bone formation and is involved in osteoblast metabolism. Therefore, when bone metabolism is rapid, such as in conditions with metabolic bone disease, the activity of osteoblasts increases with excessive osteoclast activity, and the concentration of ALP in the serum increases (Kuo and Chen, 2017; Kim M. et al., 2021). The ALP level in the OVX group increased compared to that in the sham group and decreased significantly in the SR-L group. In addition, the SR-H group had decreased ALP levels, but the decrease was not significant. These results suggest that the SR group had a reduced increase in ALP levels due to excessive osteoclast activity. AST and ALT are the most commonly used indicators of hepatotoxicity. High serum levels of AST and ALT indicate damage to hepatocytes (Ozer et al., 2008). In this study, ALT and AST were significantly decreased in the E_2_, ALN, SR-L and SR-H groups. These results indicate that the E_2_, ALN, SR-L and SR-H groups did not exhibit hepatotoxicity.

Quantitative evaluation of three-dimensional (3D) trabecular structural properties may improve the ability to understand the pharmaceutical properties of osteoporosis and to estimate bone properties. Micro-CT is a commonly used technique to understand the structural properties of bones (Jiang et al., 2000; Genant et al., 2008). BMD is an important indicator used to evaluate bone quality. In this study, micro-CT was used to analyze the trabecular bone structure in rats. BV/TV is the area ratio of trabecular bone in the area of interest. Tb.Th is the average thickness of the trabecular trabeculae. Tb. Sp is the average distance of trabecular trabeculae (Small, 2005). In this study, BMD, BV/TV and Tb.Th were significantly decreased in the OVX group compared to the sham group. The E_2_, ALN and SR groups had increased BMD, BV/TV and Tb.Th values compared with the OVX group. Tb. sp was significantly increased in the OVX group compared to the sham group. E_2_, ALN and SR increased Tb. sp. in the OVX-induced rat model. Our data showed that SR significantly inhibited bone loss in the OVX-induced rat model.

Trabecular area and bone quality reduction is a common symptom in patients with osteoporosis. Therefore, the trabecular area was used as an important indicator of antiosteoporotic activity (Osterhoff et al., 2016). The OVX group had a decreased trabecular area compared with the sham group, and the OVX-induced reduced trabecular area was increased in the E_2_, ALN and SR groups. Since estrogen deficiency through OVX causes abnormal activity of osteoclasts, TRAP staining in the femur is used to investigate the antiosteoporosis effect in various studies (Lee et al., 2019). In this study, the OVX group increased the number and area of TRAP-positive cells compared to the sham group, and SR inhibited this increase. The role of estradiol deficiency on osteoblast activation has been discussed in various researches. Confirming the study of Li et al., osteoblast activity was increased after OVX compared to the sham group (Li et al., 2019). Since it was found that the expression of estrogen suppresses the death of osteoblasts and prolongs the lifespan of osteoblasts, it is believed that the lack of estrogen due to menopause accelerates the death of osteoblasts (Bradford et al., 2010). Similar to Li’s study, our results showed that osteoblast activity was decreased in the OVX group compared to the sham group, and the osteoblast activity was significantly increased in the SR group than the OVX group. The result of this study is that SR suppresses the decrease in osteoblast activity caused by estradiol deficiency, and it is considered that SR plays a positive role in osteoblast differentiation and activity, as in the cellular level results.

Immunohistochemical (IHC) staining is a staining method used to detect the presence of specific protein markers (Duraiyan et al., 2012; Kim et al., 2020; Kim M. et al., 2021). In our study, the number of CTK-, NFATc1- and BMP-2-positive cells was evaluated by IHC staining. We found that the OVX group had elevated numbers of CTK- and NFATc1-positive cells in the OVX-induced rat model, while E_2_, ALN and SR treatment reduced their expression levels. In addition, the number of BMP-2-positive cells was decreased due to OVX, and it was confirmed that this expression was increased through E_2_, ALN and SR treatment. These experimental results indicated that SR inhibits osteoclast differentiation and increases osteoblast activity in the OVX-induced osteoporosis model, and this effect is mediated by NFATc1 and BMP-2 similar to the *in vitro* experimental results.

The present study had several limitations. 1) Osteoporosis occurs for various reasons, including menopause, aging, inflammation, and steroid overdose (Sozen et al., 2017). In this study, only the effects of SR on postmenopausal osteoporosis were verified. In particular, the osteoblast differentiation-promoting effect of SR is expected to be effective in senile osteoporosis caused by metabolic deterioration due to aging. In the future, it will be expected to have research value to investigate the anti-osteoporosis effect of SR in aged rat or mice with an aging phenotype such as senescence-accelerated mouse prone 6 (SAMP6) (Azuma et al., 2018). 2) Another limitation is that the mechanism analysis of osteoclasts and osteoblasts has been confined. Various previous studies have demonstrated that the TRAF6/MAPK/NF-κB/NFATc1/c-Fos mechanism is representative as the mechanism for inducing osteoclast differentiation (Wong et al., 1998; Mizukami et al., 2002). However, including this, the mTOR/Akt mechanism involved in osteoclast survival (Tiedemann et al., 2017) and the OSCAR-stimulated calcium signaling mechanism were also found to be involved in the differentiation and activity of osteoclasts (Barrow et al., 2011). However, in this study, the pharmacological effects of SR on these mechanisms were not investigated. In addition, as for the osteoblast differentiation mechanism, various intracellular mechanisms such as Wnt/β-catenin canonical mechanisms exist (Pai et al., 2017), but this study only investigated the effect of SR on the BMP-2/SMAD mechanism and the expression of RUNX2. It is considered that the limitations of this study occurred during the initial construction stage of the experiment. We do not know the effect of SR on some of the mechanisms because we selected and conducted experiments on mechanisms that were found to play a major role in the differentiation/activation of osteoclasts and osteoblasts through preliminary investigation. In the future, if the role of SR on the expression of all factors in osteoclasts and osteoblasts is identified through experiments such as bioinformatic analysis, it is expected that it will be helpful in understanding the mechanism of bone metabolism control of SR. 3) Antagonists are useful in explaining the mechanism of each cell and have been utilized in various studies. For example, OPG and denosumab are representative antagonists that inhibit osteoclast differentiation by regulating RANKL (Schieferdecker et al., 2014) and, noggin and chordin are antagonists that inhibit osteoblast activity by inhibiting the BMP-2 mechanism (Glister et al., 2018), These antagonists are useful in explaining the mechanism of each cell and have been utilized in various studies (Gu et al., 2016; Kim JH. et al., 2021). However, this study did not include antagonist-related studies. Our results in osteoclast experiments do not appear to inhibit osteoclast differentiation by directly binding and then inactivating RANKL in SR. SR down-regulates NFATc1/c-Fos signaling activated after RANKL-RANK binding and activates osteoclast differentiation and inhibition. Therefore, it is expected that the use of antagonists will not be of great help in the mechanism analysis. However, the role of SR in upregulating the BMP-2 mechanism is thought to be useful to explain and understand such a mechanism if an in-depth study was conducted using noggin (antagonist of bmp-2). In the future, further study of SR using these antagonists is considered as a meaningful study that can more clearly explain the mechanism of action of osteoclasts and osteoblasts in SR. iv) The cellular concentration of SR used in this study was determined by referring to the toxicity verification through CCK-8 and previous study (Liu et al., 2020). However, these concentrations are a high concentration relative to the concentration in the consensus document of ethnopharmacology (Heinrich et al., 2020). Because this may introduce artefacts in the model used, it is recommended to study appropriate dose levels in future studies.

## Conclusion

In summary, SR inhibited osteoclast differentiation, function, and bone resorption through the TRAF6/MAPK/NF-κB/NFATc1/c-Fos pathways and stimulates osteoblast differentiation by increased protein expression of the BMP-2/samd signaling pathway. Moreover, SR protected against bone loss in OVX-induced rats. However, the concentration used in the verification test of SR’s osteoclast inhibitory ability is likely to produce artefacts, so it is recommended to study the appropriate drug dose level reflecting the drug-extract ratio and other basic pharmaceutical parameters in the future. Nevertheless, our results appear to advance our knowledge of SR and successfully demonstrate its potential role as a osteoclastogenesis-inhibiting and osteogenesis-promoting herbal medicine for the treatment of postmenopausal osteoporosis.

## Data Availability

The original contributions presented in the study are included in the article/Supplementary Material, further inquiries can be directed to the corresponding authors.

## Abbreviations

ALP, alkaline phosphatase; ALT, alanine transferase; AST, aspartate aminotransferase; ATP6v0d2, ATPase H+ transporting v0 subunit d2; BMD, bone mineral density; BMP-2, bone morphogenetic protein 2; BV/TV, trabecular bone volume; CA2, carbonic anhydrase II; DC-STAMP, dendritic cell-specific transmembrane protein; H&E, hematoxylin and eosin; IHC, immunohistochemical; LC-MS, liquid chromatography–mass spectrometry; MAPK, mitogen-activated protein kinase; MMP-9, matrix metalloproteinase-9; NFATc1, nuclear factor of activated T cells 1; NF-κB, nuclear factor-κB; OSCAR, osteoclast-associated receptor; OVX, ovariectomized; RANKL, receptor activator of nuclear factor-κB ligand; RT-PCR, real-time polymerase chain reaction; RUNX-2, runt-related transcription factor 2; SERMs, selective estrogen receptor modulators; SR, Sparganii Rhizoma; Tb. sp, trabecular separation; Tb.N, trabecular number; Tb.Th, trabecular thickness; TRAP, tartrate-resistant acid phosphatase.
