# Supplementary material for: Effects of Sparganii Rhizoma on Osteoclast Formation and Osteoblast Differentiation and on an OVX-Induced Bone Loss Model
Source: Front Pharmacol. 2022 Jan 4;12:797892. doi: 10.3389/fphar.2021.797892 (PMC8764242; doi:10.3389/fphar.2021.797892)
Supplement: Supplementary file 1 [file Presentation1.pptx]

## Slide 1
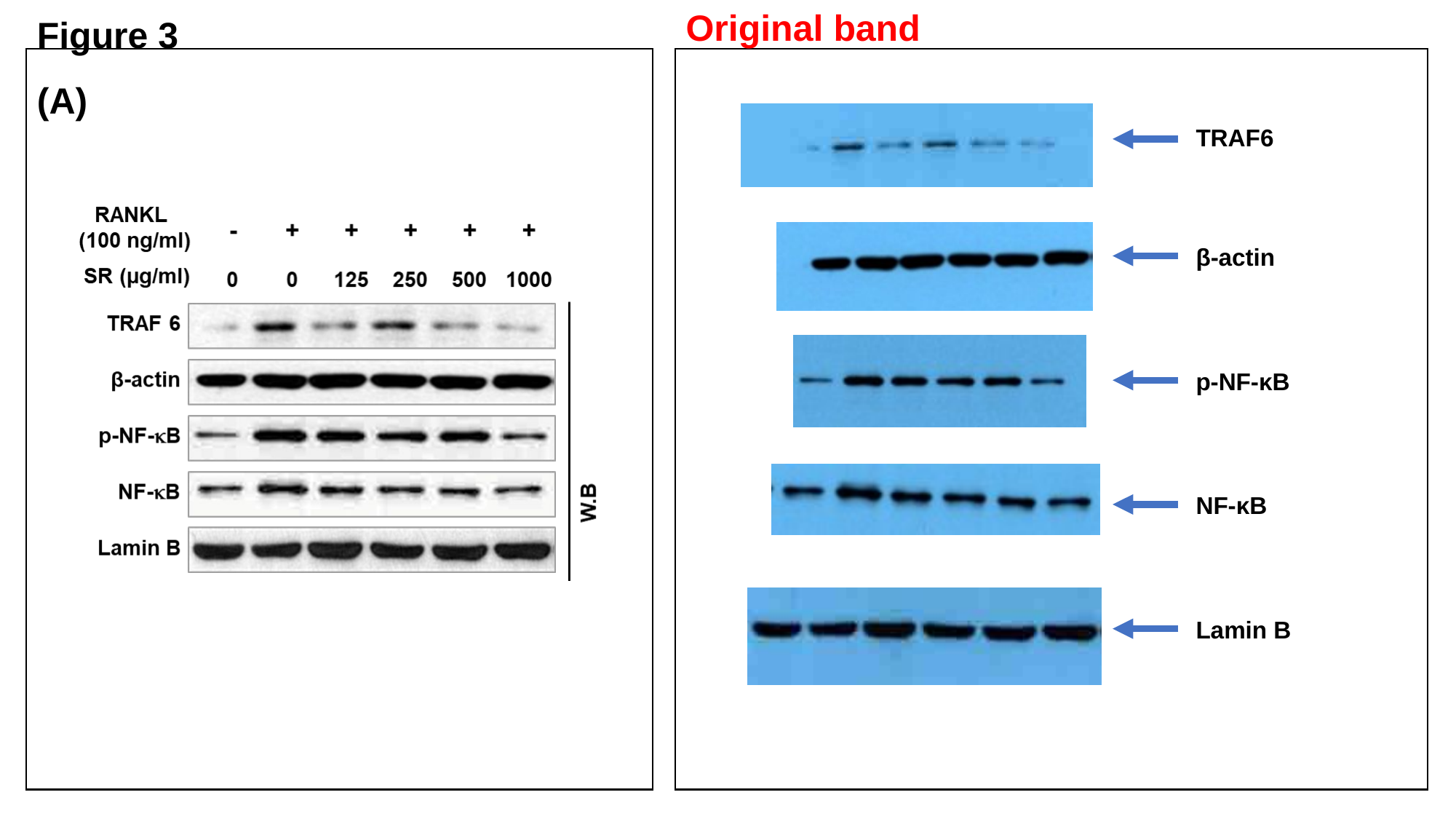

Figure 3 (A)
Original band
TRAF6
β-actin
p-NF-κB
NF-κB
Lamin B

## Slide 2
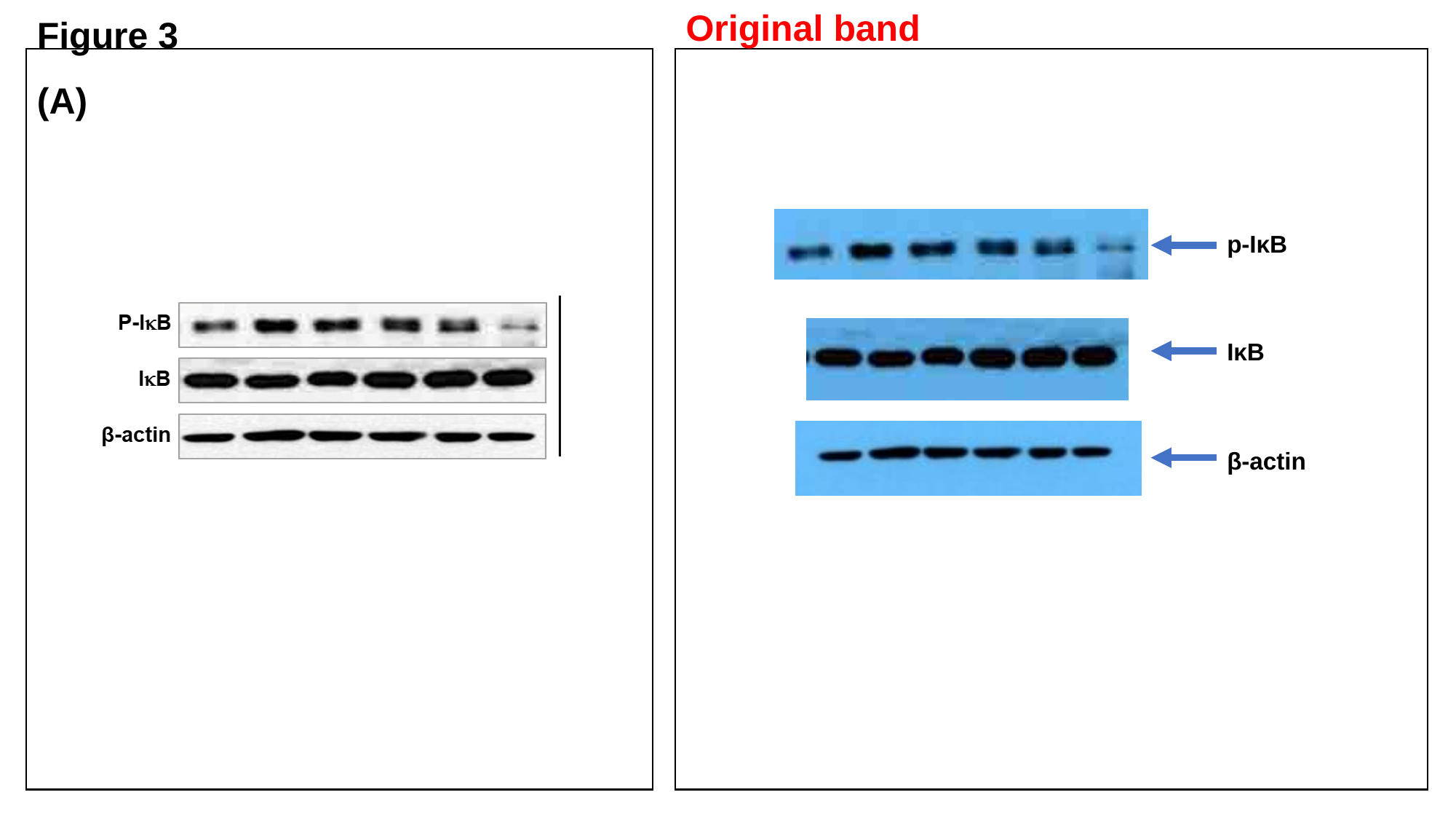

Figure 3 (A)
Original band
p-IκB
IκB
β-actin

## Slide 3
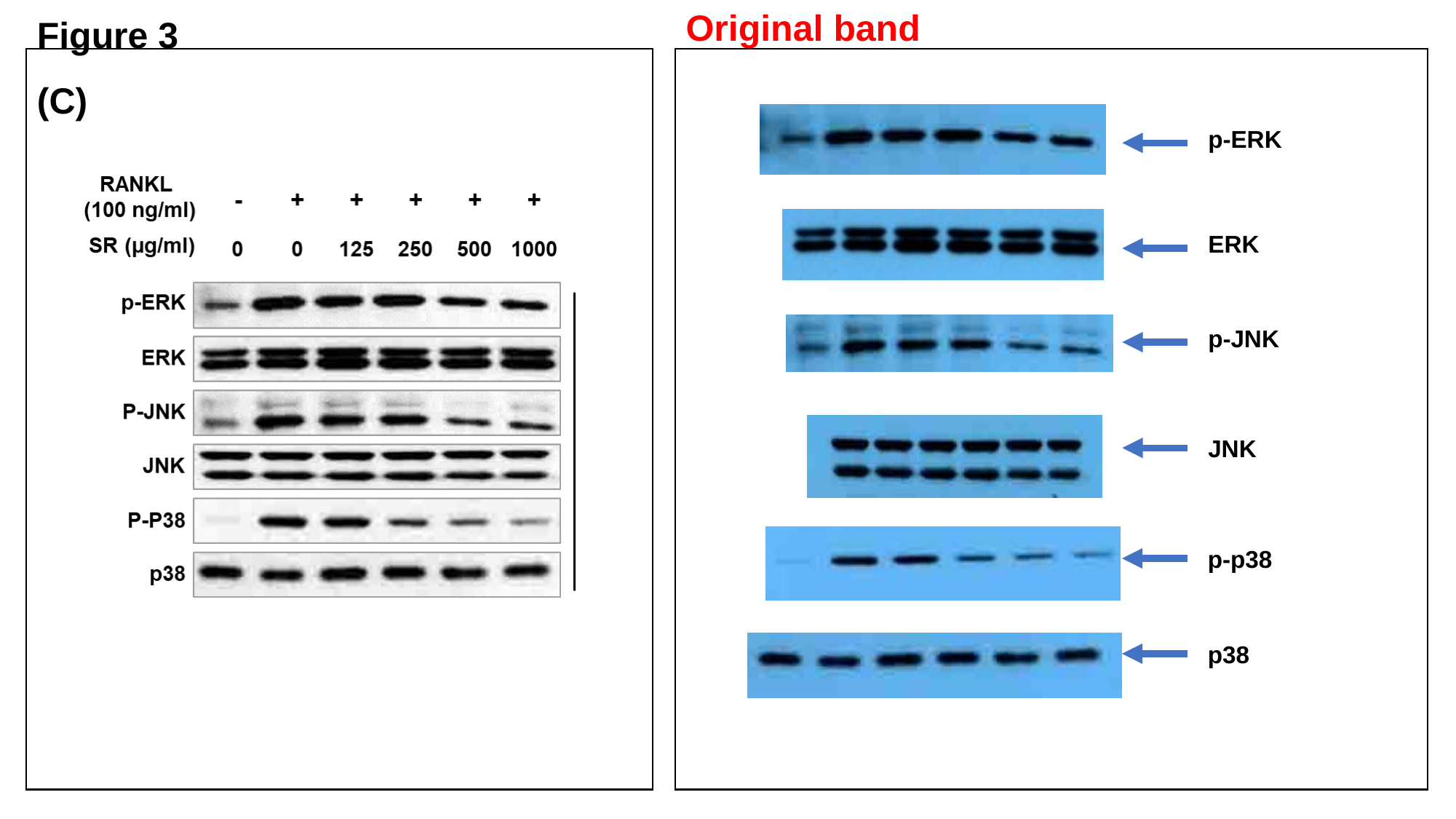

Figure 3 (C)
Original band
p-ERK
ERK
p-JNK
JNK
p-p38
p38

## Slide 4
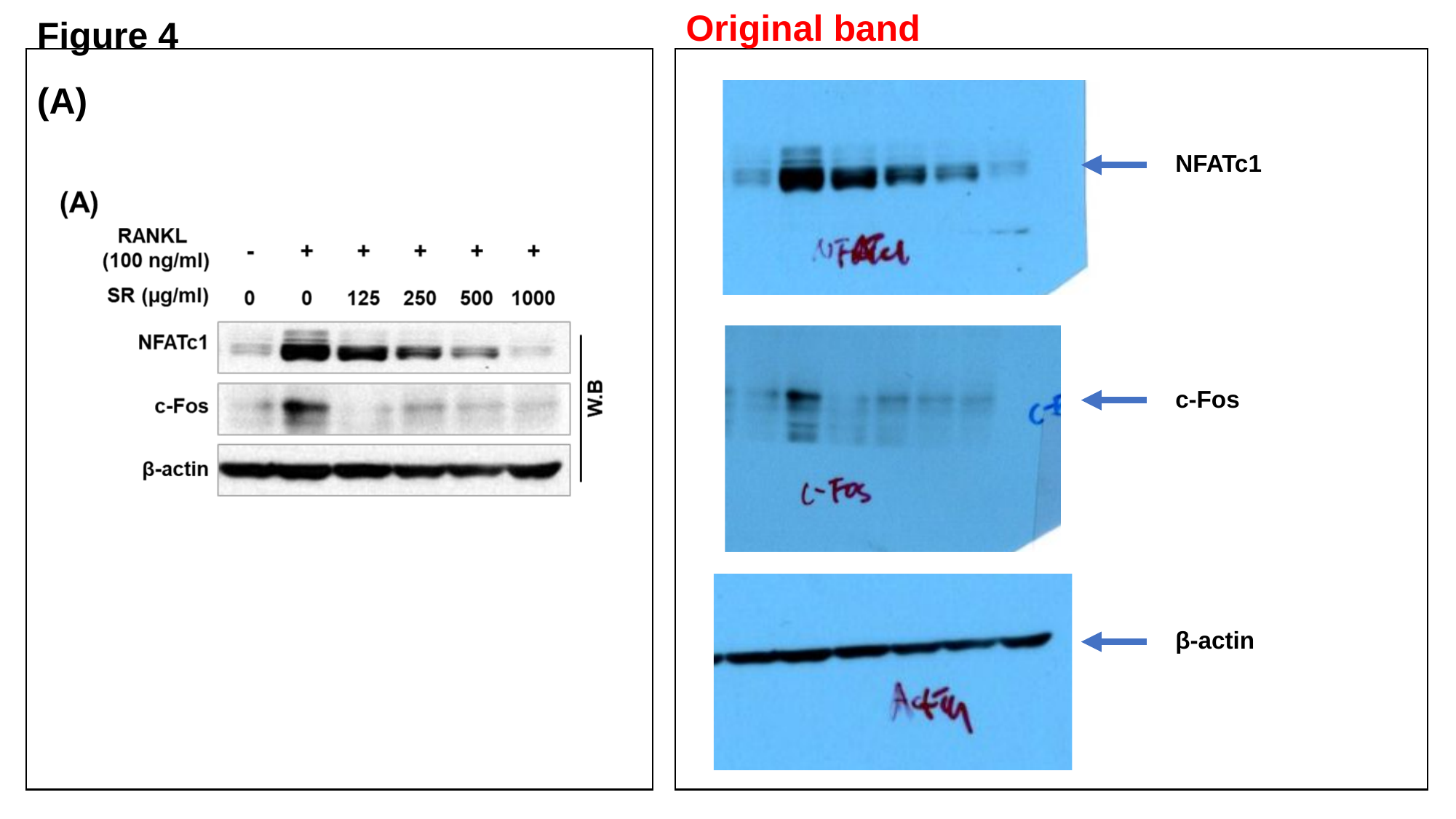

Figure 4 (A)
Original band
NFATc1
c-Fos
β-actin

## Slide 5
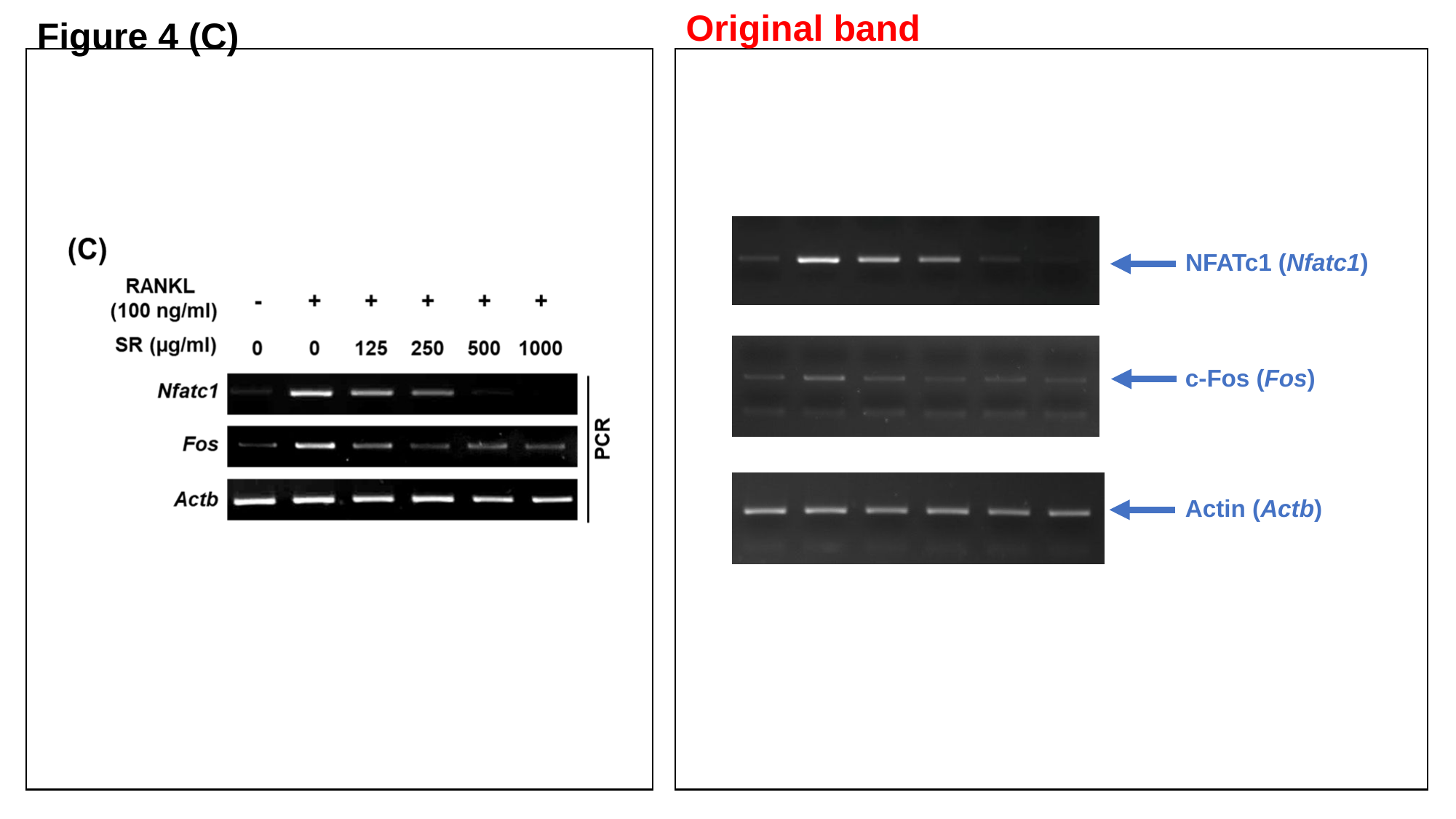

Figure 4 (C)
Original band
NFATc1 (Nfatc1)
c-Fos (Fos)
Actin (Actb)

## Slide 6
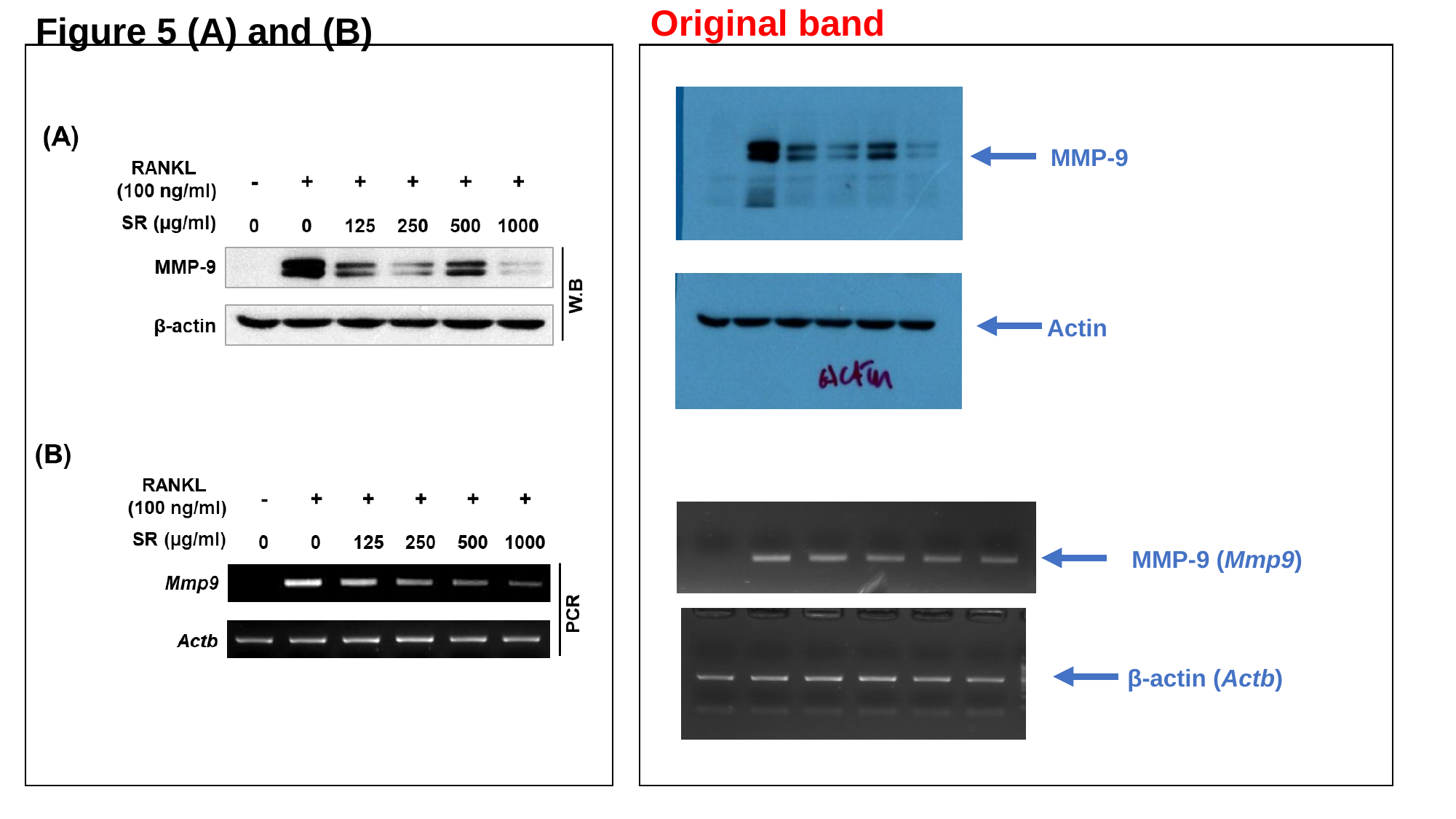

Figure 5 (A) and (B)
Original band
바꾸기
MMP-9
Actin
MMP-9 (Mmp9)
β-actin (Actb)

## Slide 7
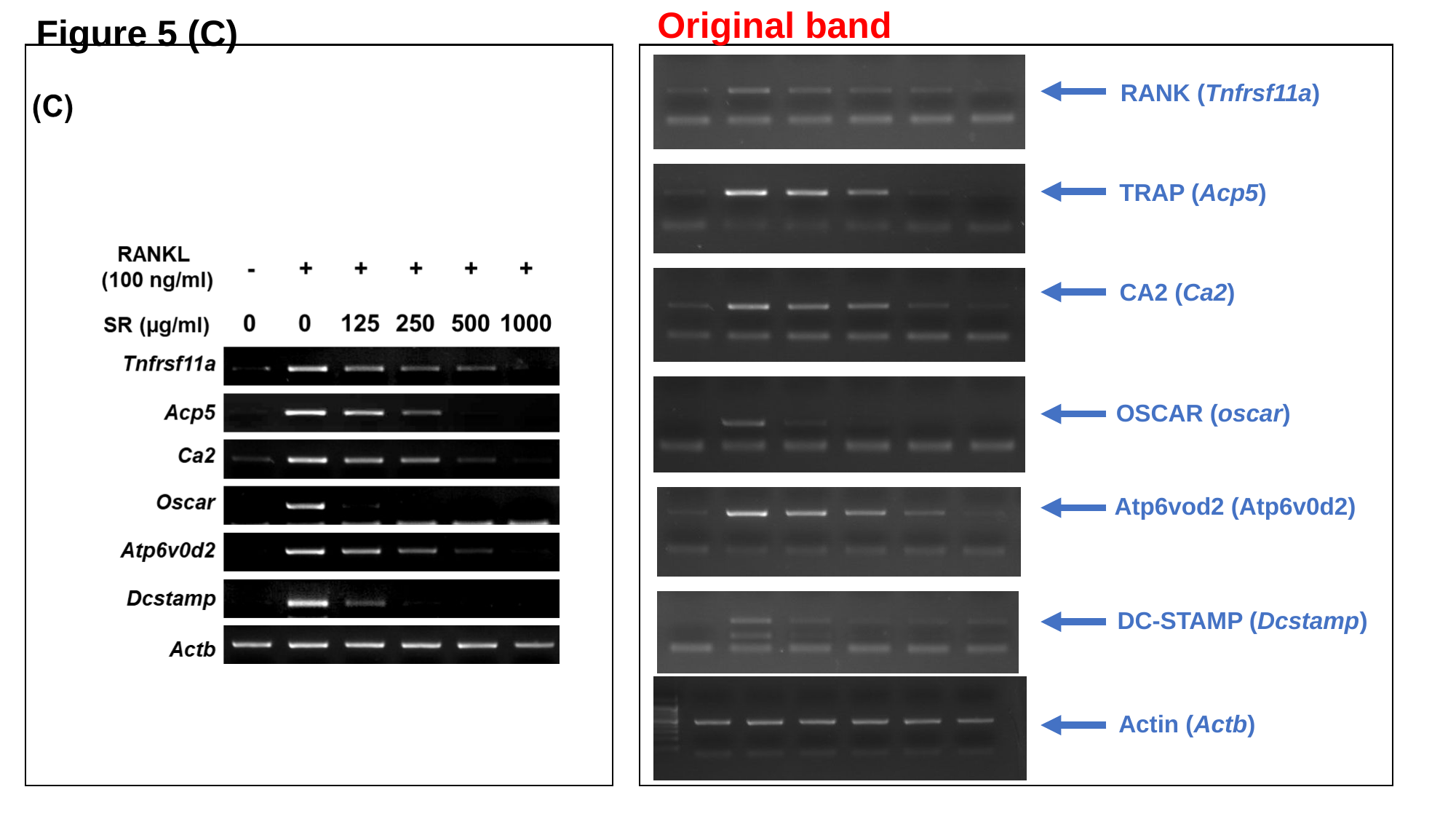

Figure 5 (C)
Original band
RANK (Tnfrsf11a)
TRAP (Acp5)
CA2 (Ca2)
OSCAR (oscar)
Atp6vod2 (Atp6v0d2)
DC-STAMP (Dcstamp)
Actin (Actb)

## Slide 8
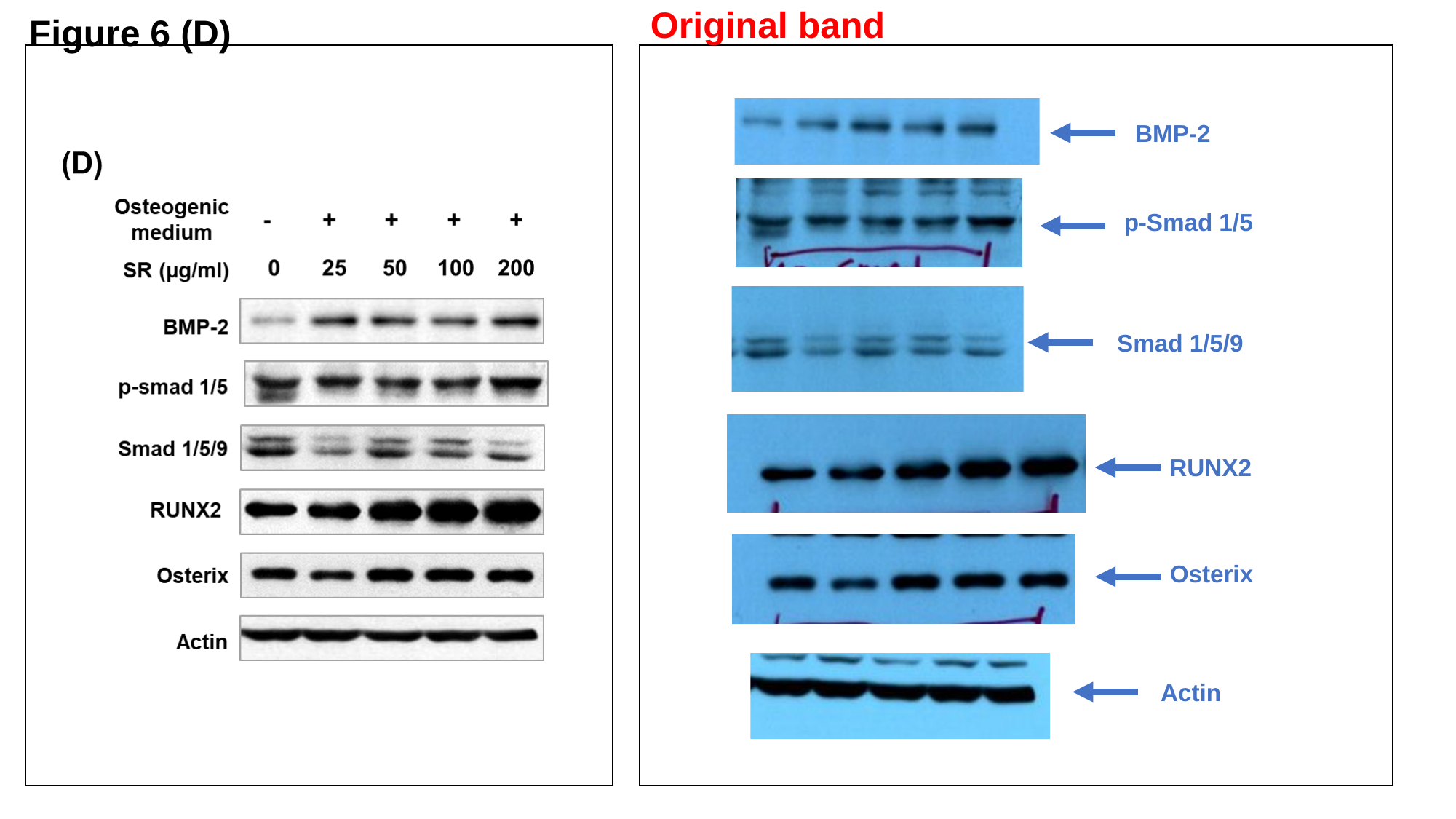

Figure 6 (D)
Original band
BMP-2
p-Smad 1/5
Smad 1/5/9
RUNX2
Osterix
Actin

## Slide 9
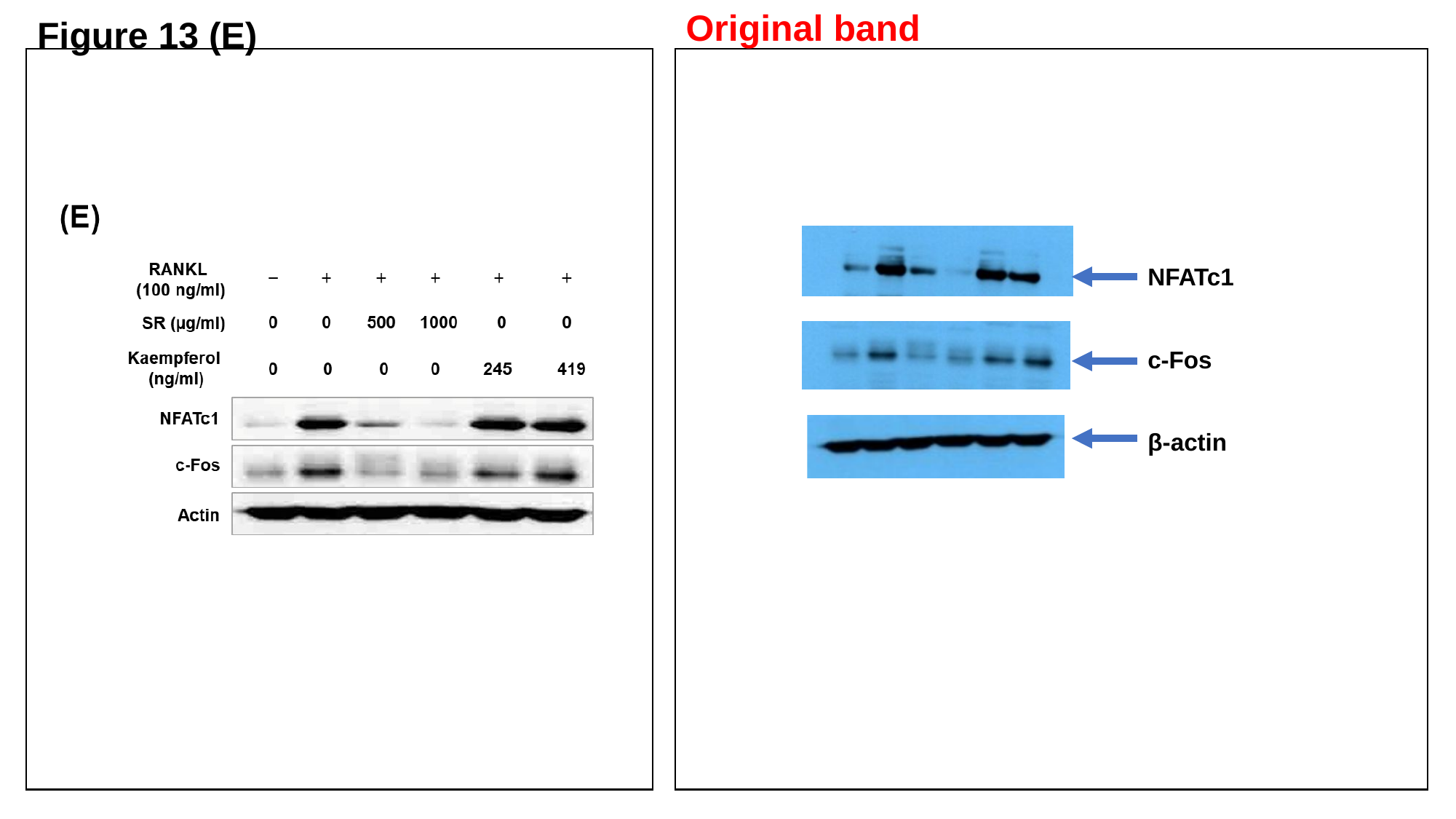

Figure 13 (E)
Original band
NFATc1
c-Fos
β-actin

## Slide 10
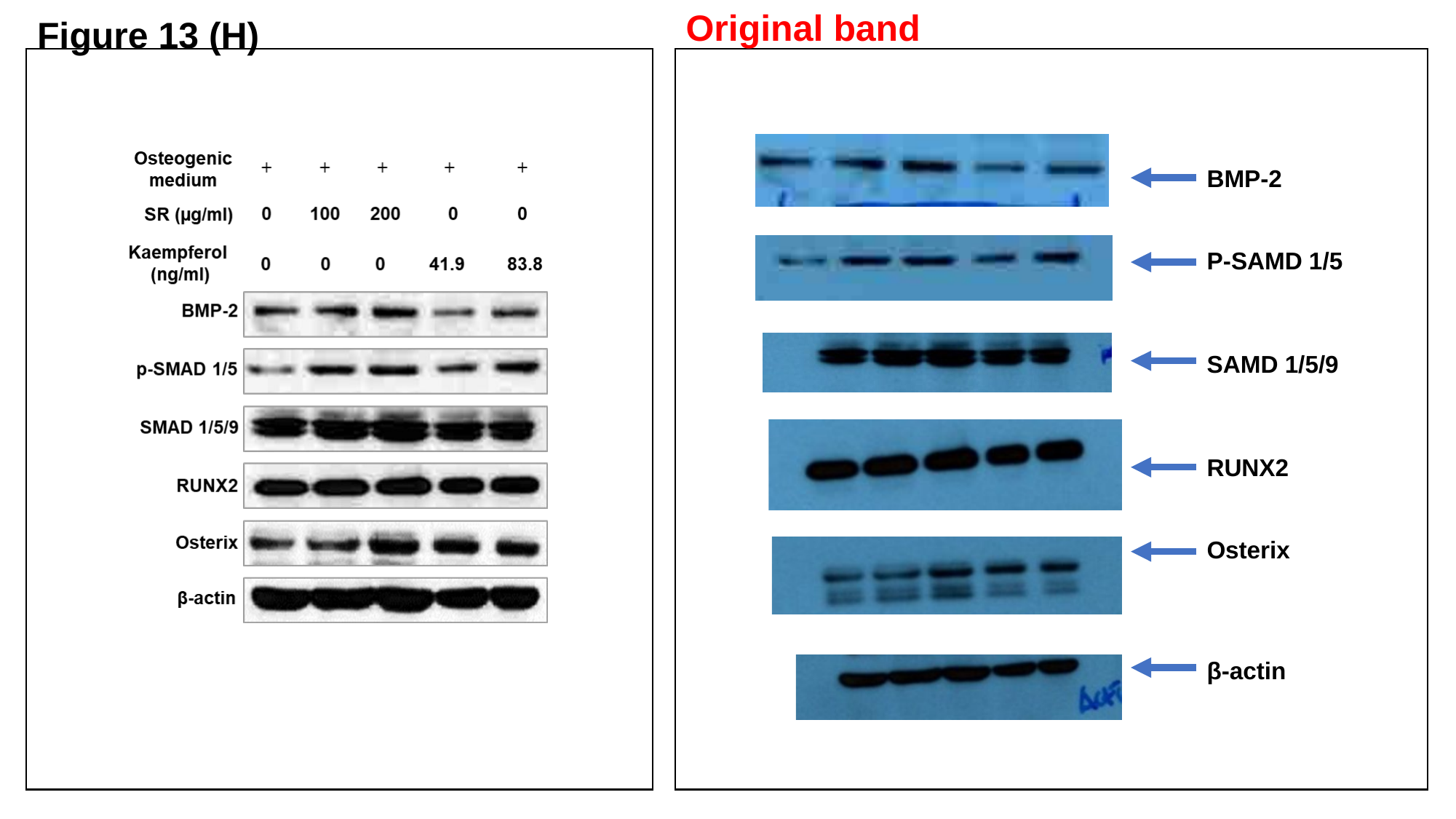

Figure 13 (H)
Original band
BMP-2
P-SAMD 1/5
SAMD 1/5/9
RUNX2
Osterix
β-actin
