# Supplementary material for: Effects of Sparganii Rhizoma on Osteoclast Formation and Osteoblast Differentiation and on an OVX-Induced Bone Loss Model
Source: Front Pharmacol. 2022 Jan 4;12:797892. doi: 10.3389/fphar.2021.797892 (PMC8764242; doi:10.3389/fphar.2021.797892)
Supplement: Supplementary file 2 [file Presentation2.PPTX]

## Slide 1
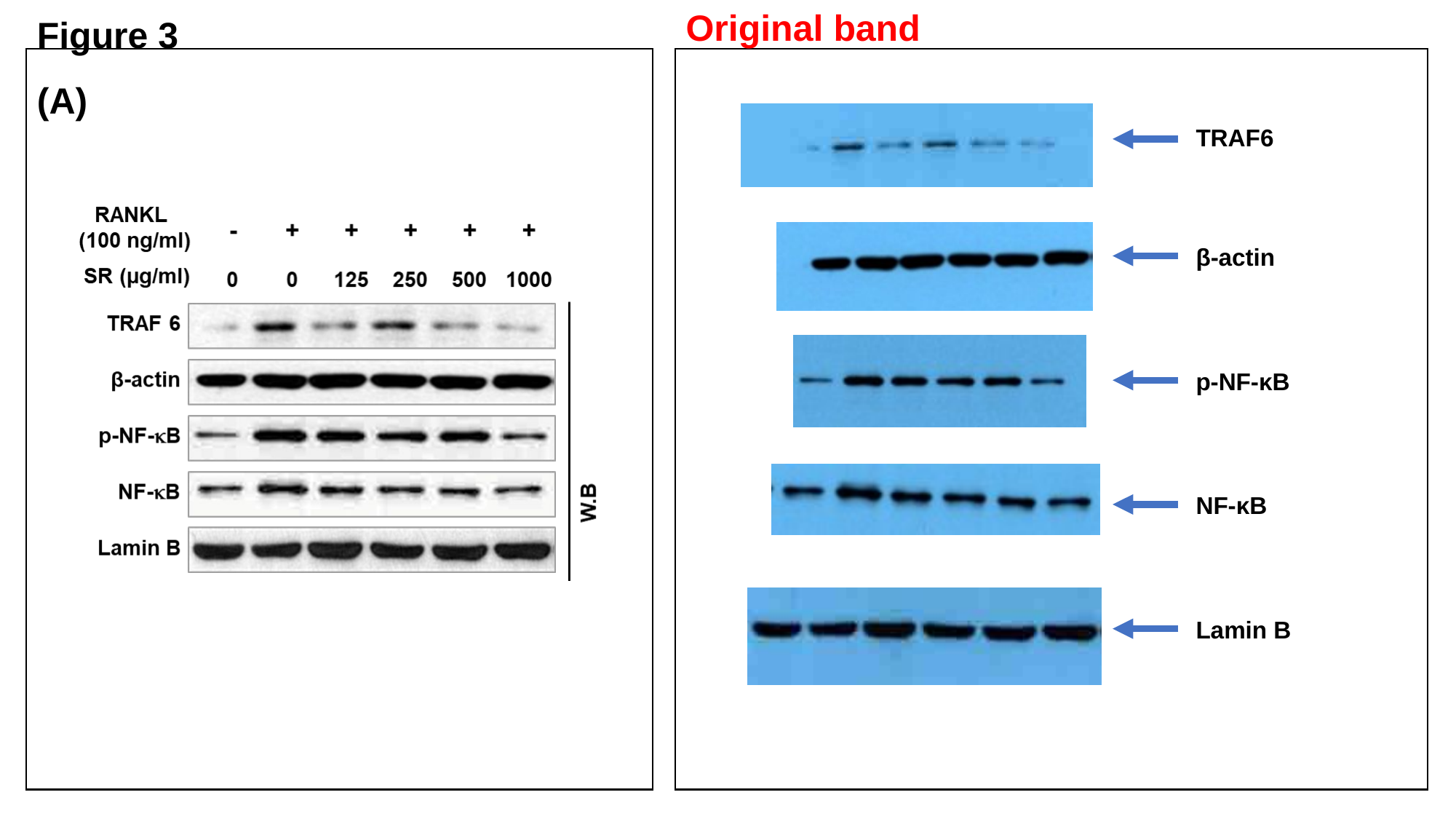

Figure 3 (A)
Original band
TRAF6
β-actin
p-NF-κB
NF-κB
Lamin B

## Slide 2
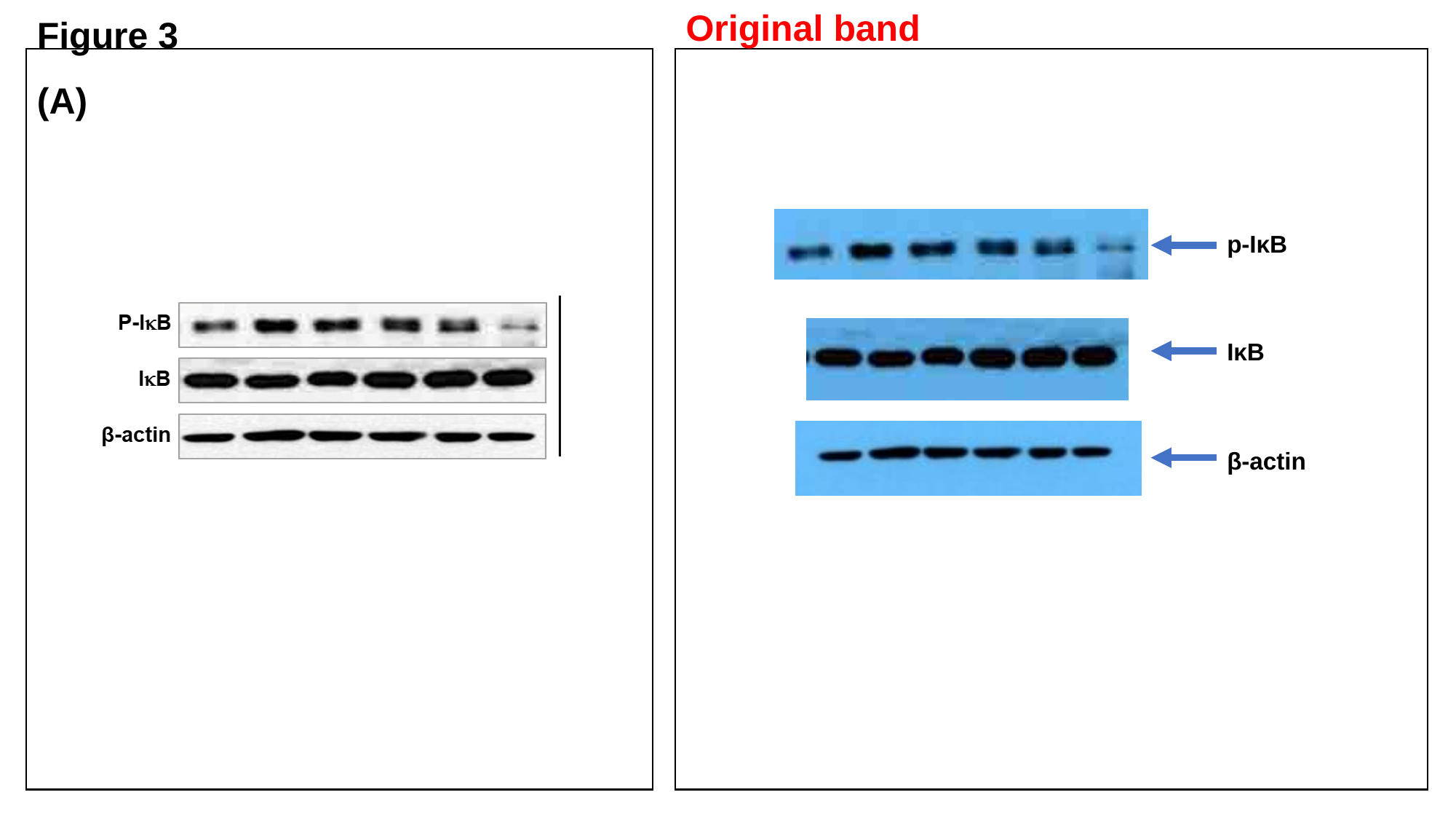

Figure 3 (A)
Original band
p-IκB
IκB
β-actin

## Slide 3
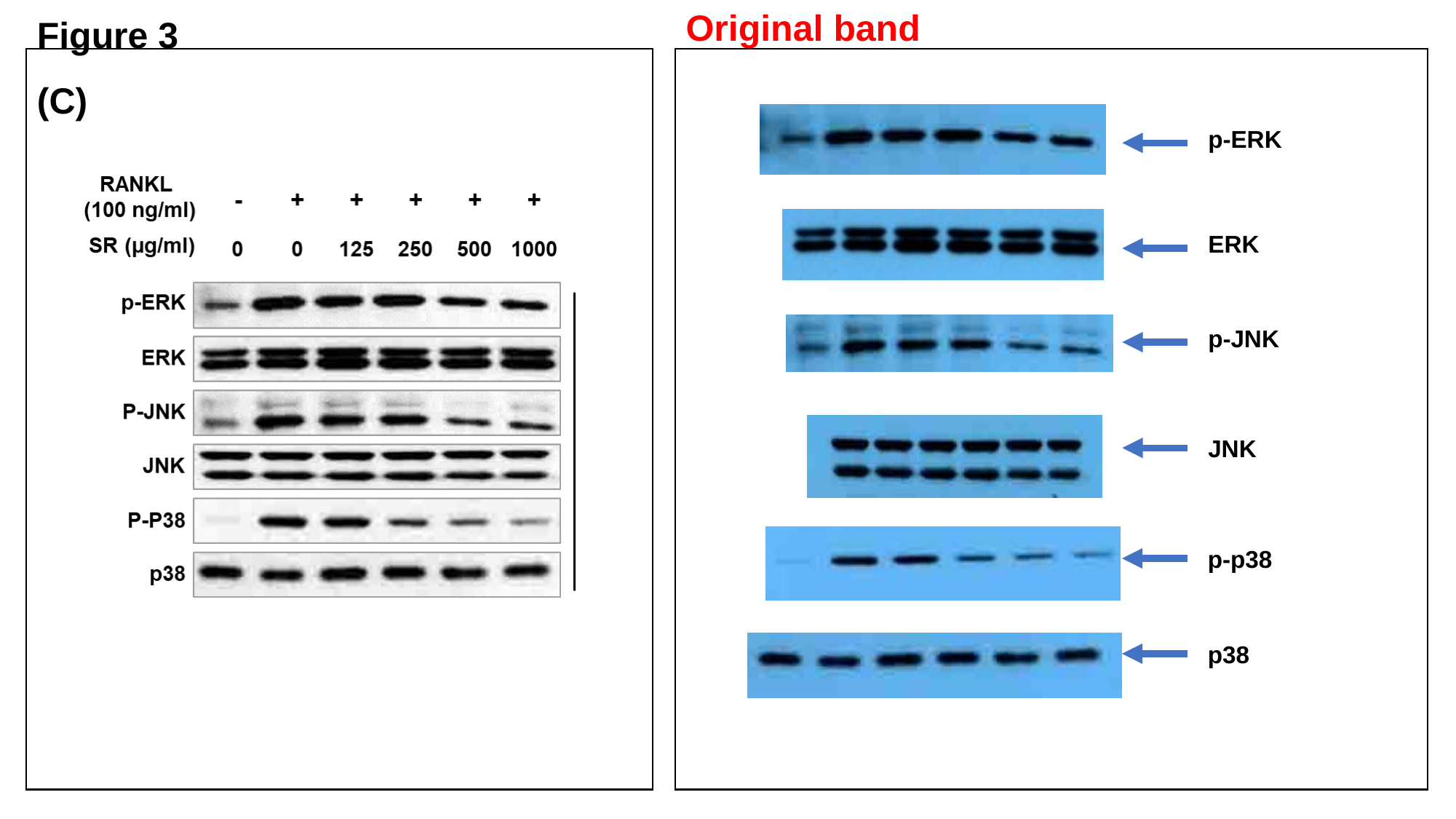

Figure 3 (C)
Original band
p-ERK
ERK
p-JNK
JNK
p-p38
p38

## Slide 4
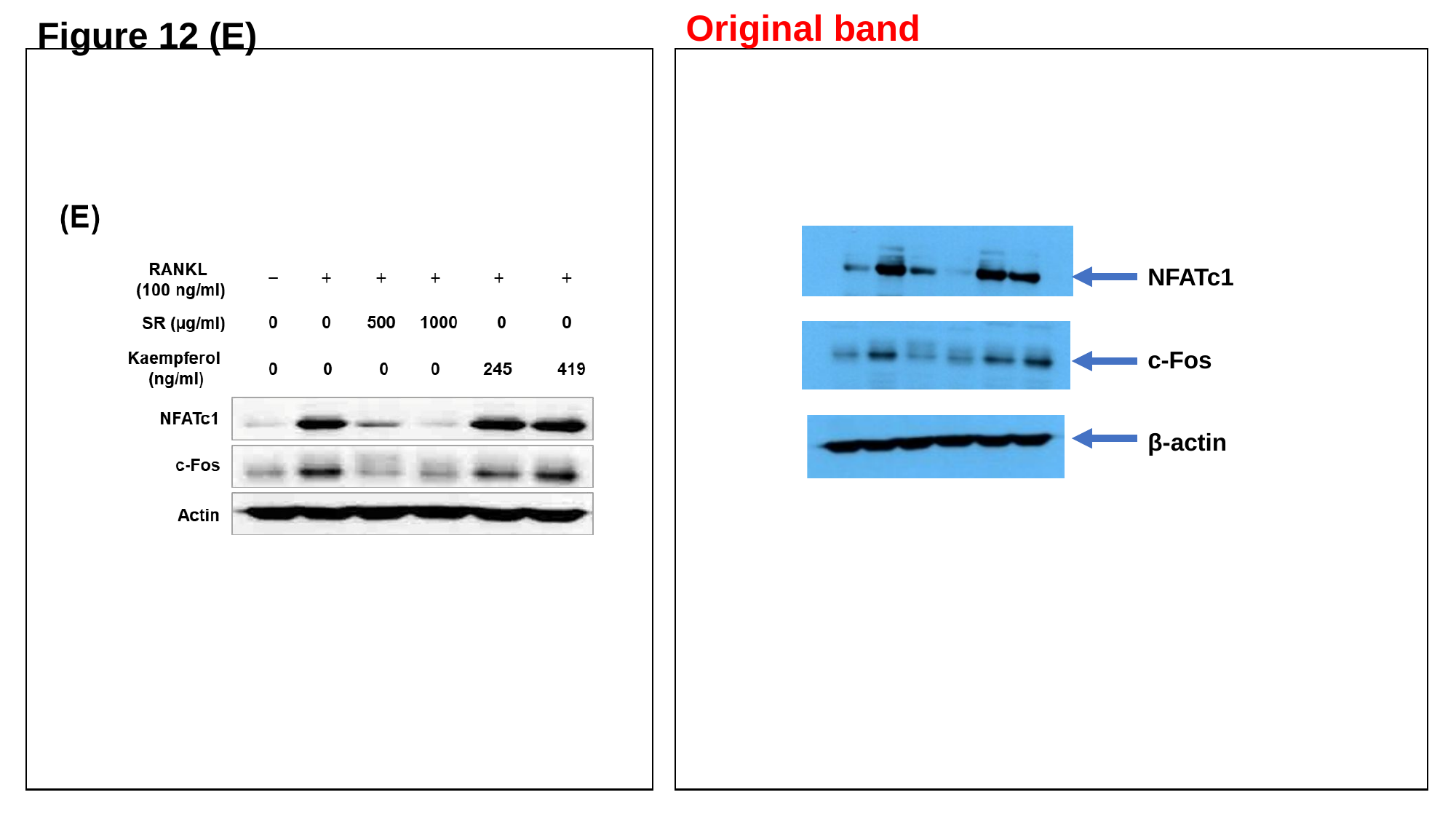

Figure 12 (E)
Original band
NFATc1
c-Fos
β-actin

## Slide 5
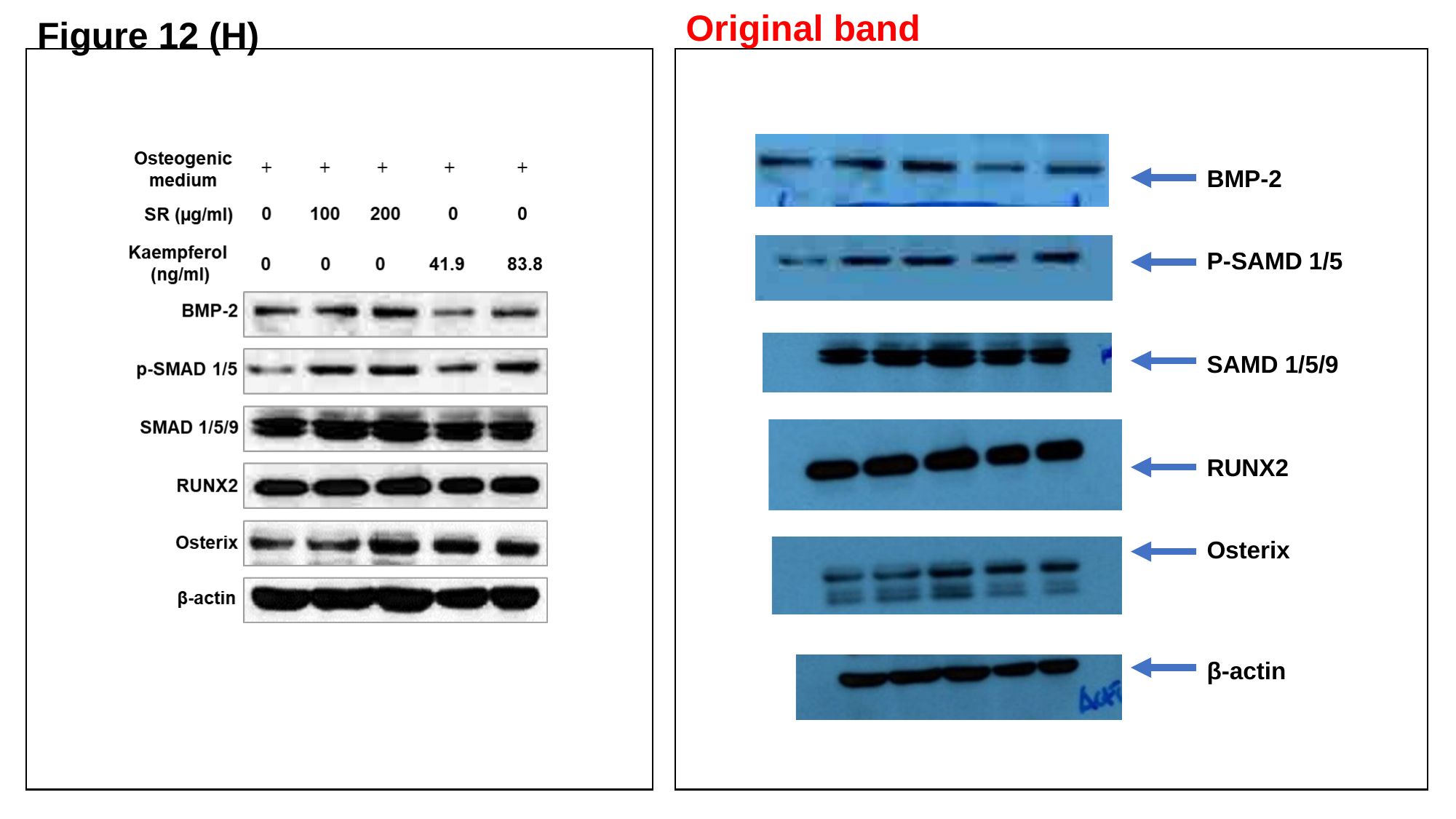

Figure 12 (H)
Original band
BMP-2
P-SAMD 1/5
SAMD 1/5/9
RUNX2
Osterix
β-actin
